# Enhancement of radiosensitivity by the novel anticancer quinolone derivative vosaroxin in preclinical glioblastoma models

**DOI:** 10.18632/oncotarget.16168

**Published:** 2017-03-13

**Authors:** Giovanni Luca Gravina, Andrea Mancini, Claudia Mattei, Flora Vitale, Francesco Marampon, Alessandro Colapietro, Giulia Rossi, Luca Ventura, Antonella Vetuschi, Ernesto Di Cesare, Judith A. Fox, Claudio Festuccia

**Affiliations:** ^1^ Department of Biotechnological and Applied Clinical Sciences, Division of Radiotherapy, University of L’Aquila, L’Aquila, Italy; ^2^ Department of Biotechnological and Applied Clinical Sciences, Laboratory of Radiobiology, University of L’Aquila, L’Aquila, Italy; ^3^ Department of Biotechnological and Applied Clinical Sciences, Laboratory of Neurosciences, University of L’Aquila, L’Aquila, Italy; ^4^ Department of Biotechnological and Applied Clinical Sciences, Chair of Human Anatomy, University of L’Aquila, L’Aquila, Italy; ^5^ Sunesis Pharmaceuticals Inc., South San Francisco, CA, USA

**Keywords:** glioblastoma, topoisomerase II, vosaroxin, double-strand breaks, radiotherapy

## Abstract

**Purpose:**

Glioblastoma multiforme (GBM) is the most aggressive brain tumor. The activity of vosaroxin, a first-in-class anticancer quinolone derivative that intercalates DNA and inhibits topoisomerase II, was investigated in GBM preclinical models as a single agent and combined with radiotherapy (RT).

**Results:**

Vosaroxin showed antitumor activity in clonogenic survival assays, with IC_50_ of 10−100 nM, and demonstrated radiosensitization. Combined treatments exhibited significantly higher γH2Ax levels compared with controls. In xenograft models, vosaroxin reduced tumor growth and showed enhanced activity with RT; vosaroxin/RT combined was more effective than temozolomide/RT. Vosaroxin/RT triggered rapid and massive cell death with characteristics of necrosis. A minor proportion of treated cells underwent caspase-dependent apoptosis, in agreement with *in vitro* results. Vosaroxin/RT inhibited RT-induced autophagy, increasing necrosis. This was associated with increased recruitment of granulocytes, monocytes, and undifferentiated bone marrow–derived lymphoid cells. Pharmacokinetic analyses revealed adequate blood-brain penetration of vosaroxin. Vosaroxin/RT increased disease-free survival (DFS) and overall survival (OS) significantly compared with RT, vosaroxin alone, temozolomide, and temozolomide/RT in the U251-luciferase orthotopic model.

**Materials and Methods:**

Cellular, molecular, and antiproliferative effects of vosaroxin alone or combined with RT were evaluated in 13 GBM cell lines. Tumor growth delay was determined in U87MG, U251, and T98G xenograft mouse models. (DFS) and (OS) were assessed in orthotopic intrabrain models using luciferase-transfected U251 cells by bioluminescence and magnetic resonance imaging.

**Conclusions:**

Vosaroxin demonstrated significant activity *in vitro* and *in vivo* in GBM models, and showed additive/synergistic activity when combined with RT in O6-methylguanine methyltransferase-negative and -positive cell lines.

## INTRODUCTION

Glioblastoma multiforme (GBM) is an aggressive brain tumor associated with invasive behavior, high rate of recurrence, and an average survival of less than 15 months, irrespective of treatment [1–3]. Histologically, malignant gliomas are characterized by hypercellularity, nuclear pleomorphism, microvascular proliferation, pseudopalisading necrosis, reactive gliosis, microglial activation, disrupted vasculature, breakdown of the blood-brain barrier, and increases in hypoxia. The high malignancy of GBM is due to diffuse infiltration into the brain, high resistance to apoptosis [4], robust angiogenesis [5], tumor cell heterogeneity [6], proliferation of cancer stem-like cells [7], and an inflammatory state that results in recruitment of circulating lymphocytes and monocytes. GBM tumors consist of a heterogeneous population of tumor cells and contain immune cells that, with tumor vasculature and the extracellular matrix, constitute the tumor microenvironment. Interactions among these different cell types and cytokines may promote tumor development and progression. Macrophages, specifically tumor-associated macrophages (TAMs), are the most common cell type among tumor-infiltrating immune cells [8, 9]. TAMs from human neoplasms express arginase 1, interleukin (IL)-10, and transforming growth factor beta (TGF-β); these cytokines reduce the antitumor activity of T cells and natural killer cells, and modulate tumor proliferation, infiltration, and angiogenesis [10]. Previous studies of TAM populations in glioma tissues have shown that activated microglia/macrophages (especially M2) express high levels of CD68, CD163, CD204, and CD206 [11]. Cells expressing monocyte and M2 markers are found dispersed throughout the tumor parenchyma.

The recent progress in the treatment of malignant gliomas is attributable to the introduction of the alkylating agent temozolomide (TMZ [12]). However, resistance to TMZ resulting from O6-methylguanine methyltransferase (MGMT) expression remains a major issue. Elevation of MGMT expression has been associated with chemoresistance in a large fraction of GBM, while the resistance mechanisms of MGMT-negative tumors are not well understood [13]. Thus, there is a clear need for effective second-line agents in patients with GBM who developed drug resistance.

Type II topoisomerases are essential for the survival of eukaryotic cells [14]. These enzymes maintain DNA topology, disentangling DNA that becomes knotted, underwound, or overwound in the process of replication, and are required to maintain correct chromosome condensation, decondensation, and segregation. Topoisomerase II is a validated target of a number of therapeutics currently in use for the treatment of diverse cancers, including intercalative topoisomerase II-poisoning drugs such as the anthracyclines (doxorubicin, daunorubicin, and idarubicin), and the anthracenedione mitoxantrone [15–19]. Several topoisomerase II inhibitors are known to potentiate the effects of radiation on tumor cells, although the mechanisms of radiation sensitization remain an area of research [20–23]. Anthracyclines have effective and broad-spectrum antitumor activity but their clinical utility is often limited by systemic toxicity (eg, cardiotoxicity with doxorubicin) or drug resistance (frequently mediated by P-glycoprotein) [18, 19, 24, 25].

**Figure 1:**
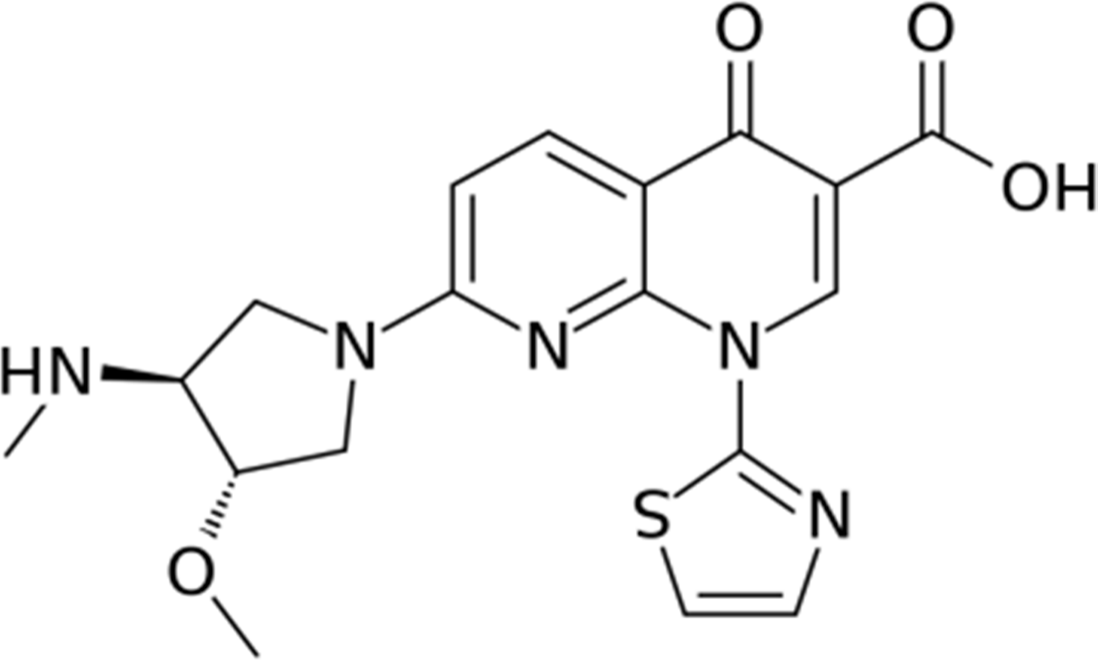
Chemical structure of vosaroxin

Vosaroxin is a naphthyridine analog (Figure 1), structurally related to quinolone antibacterials, that exerts its anticancer activity exclusively by DNA intercalation and inhibition of topoisomerase II, leading to site-selective DNA double-strand breaks and apoptosis [26–28]. Vosaroxin is not a substrate for P-glycoprotein drug pumps, and can induce apoptosis independent of p53, thereby avoiding two common mechanisms of drug resistance [29]. Vosaroxin has been shown to be active against various *in vitro* and *in vivo* tumor models including breast, bladder, pancreas, colon, ovarian, gastric, and lung cancer [29–35]. It has also shown synergistic activity with platinum agents, anthracyclines, antimetabolites, and targeted therapies in tumor models [36]. In a recently completed pivotal phase 3 study in relapsed or refractory acute myeloid leukemia (*N* = 711), no increase in organ-specific toxicities (cardiac, renal, hepatic, or pulmonary) was observed with vosaroxin/cytarabine treatment in comparison with placebo/cytarabine treatment [37]. Nonclinical studies provide supportive evidence of an absence of toxic metabolite formation [31, 38].

Previously, vosaroxin has been shown to enhance radiosensitivity in several tumor cell types, including glioma cell lines [39]; the current study confirms and extends these findings. This study assessed the effect of vosaroxin on post-irradiation sensitivity in a series of 13 glioma cell lines using clonogenic assay. Subsequent mechanistic and *in vivo* studies were performed with MGMT-negative/TMZ-sensitive (U87MG and U251) cells and MGMT-positive/TMZ-resistant (T98G) cells. *In vivo* radiosensitization was measured by subcutaneous tumor growth delay in U87MG and T98G models as well as in luciferase-transfected U251 cells injected orthotopically into the brains of female CD1 nu/nu nude mice.

## RESULTS

### Vosaroxin reduced cell viability and induced G2/M cell cycle arrest and apoptosis in glioma cell models

**Figure 2:**
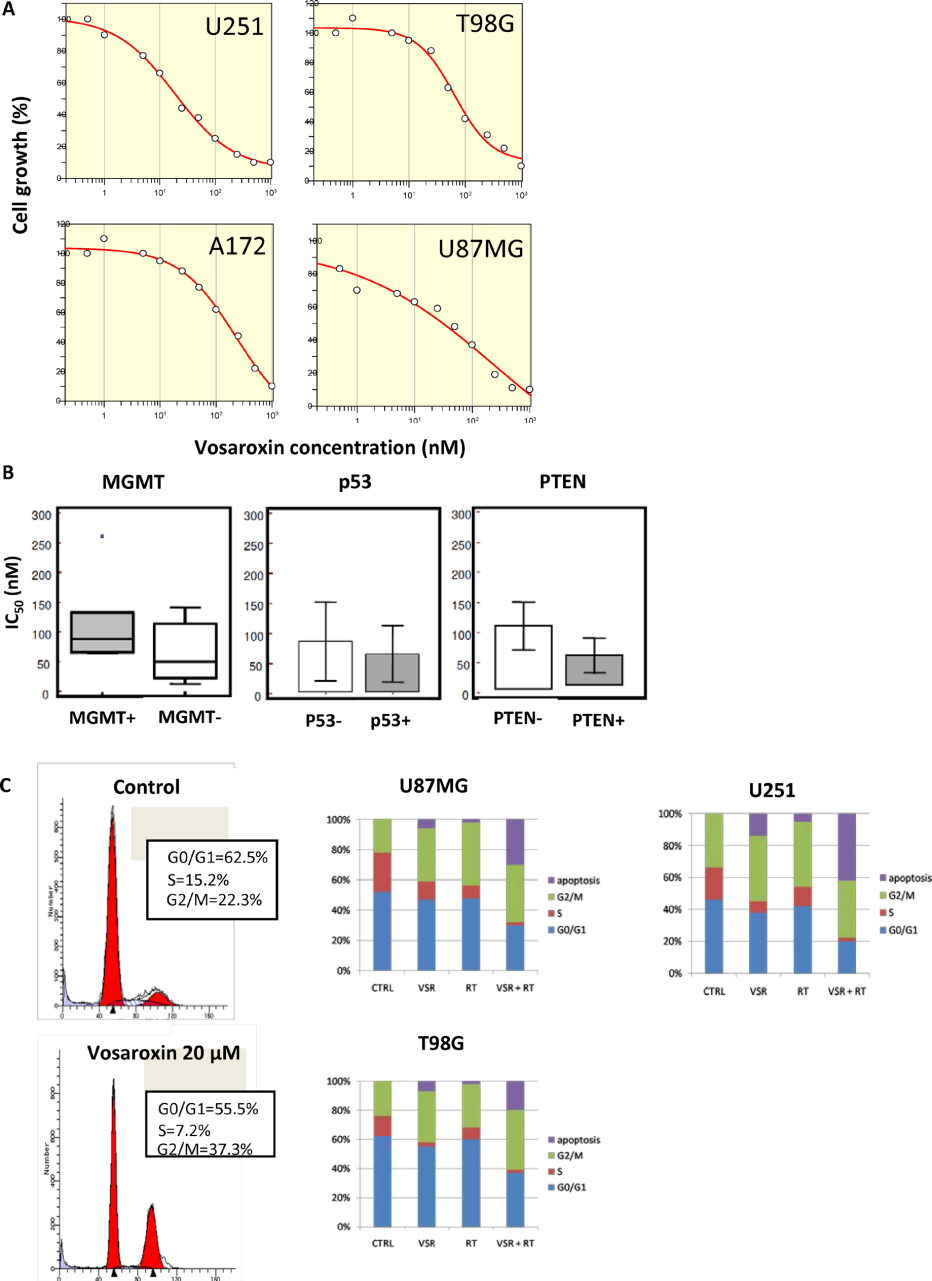
Effects of vosaroxin on glioma cell lines (**A**) Growth inhibition curves for U251, T98G, A172, and U87MG glioma cell lines treated with vosaroxin, generated with Grafit software. (**B**) No statistically significant differences were found in the IC_50_ of vosaroxin in glioma cell lines by MGMT, p53, or PTEN status. (**C**) Cell cycle analysis of vosaroxin-induced G2/M cell cycle arrest (left panels), and combination effects of vosaroxin (20 nM) and radiotherapy on cell cycle arrest and apoptosis in U87MG, U251 and T98G cells (bar graphs in right panels).

The effects of vosaroxin on cell viability were assessed in 13 human glioma cell lines and three patient-derived glioblastoma stem cell lines scored for MGMT, p53, and PTEN status (Table 1, Figure 2A). Vosaroxin demonstrated activity against all cell lines tested; 50% inhibitory concentration (IC_50_) values ranged between 12.8 nM and 260.5 nM. Interestingly, vosaroxin was found to retain its cytotoxic activity when tested against both MGMT-negative/TMZ-sensitive and MGMT-positive/TMZ-resistant cell lines (Figure 2B), in agreement with published data that suggested vosaroxin activity in multidrug-resistant (MDR) cell lines [30]. Similarly, no statistically significant differences were found by p53 or PTEN status (Figure 2B). Cell cycle analyses showed that vosaroxin induced G2/M cell cycle arrest (Figure 2C, left panels) in a dose- and time-dependent manner (data not shown). Single-agent vosaroxin showed low apoptotic-mediated cell death, but cell death increased when vosaroxin was combined with radiotherapy (RT) (Figure 2C, right panels) in U87MG, U251, and T98G cells.

**Table 1:** IC_50_ values for vosaroxin in glioma cell lines

Cell Line	IC_50_ ± SD (nM)	MGMT Status	P53 status	PTEN status
LN229	12.8 ± 2.3	Methylated	Deficient	Mutated
U251	18.6 ± 2.4	Methylated	Unfunctional	Mutated
SNB19	35.5 ± 7.0	Methylated	Unfunctional	Mutated
SF268	50.3 ± 12.0	Methylated	Unfunctional	PTEN-harboring
T98G	64.5 ± 11.0	Unmethylated	Unfunctional	PTEN-harboring
U138	65.8 ± 14.6	Unmethylated	Deficient	PTEN-harboring
U373	78.4 ± 9.7	Methylated	Unfunctional	Mutated
U118	88.5 ± 16.7	Unmethylated	Deficient	Mutated
LN18	90.0 ± 12.5	Unmethylated	Active (WT)	PTEN-harboring
A172	125.4 ± 89.4	Methylated	Active (WT)	Mutated
U87MG	140.9 ± 84.7	Methylated	Active (WT)	PTEN-deficient
SW1783	260.5 ± 9.8	Unmethylated	Unfunctional	Mutated
D54	190.0 ± 25.4	Unmethylated	Active (WT)	Wild type
BT12M	44.3 ± 5.5	Unmethylated	Active (WT)	Wild type
CSCs-5	85.4 ± 7.8	Methylated	Active (WT)	PTEN-deficient
CSCs-7	125.0 ± 15.4	Unmethylated	Unfunctional	Wild type

IC_50_: 50% inhibitory concentration; MGMT: O6-methylguanine methyltransferase; PTEN: phosphatase and tensin homolog (gene); SD: standard deviation; WT: wild type.

### Vosaroxin increased the effects of radiotherapy in glioma models *in vitro*

**Figure 3:**
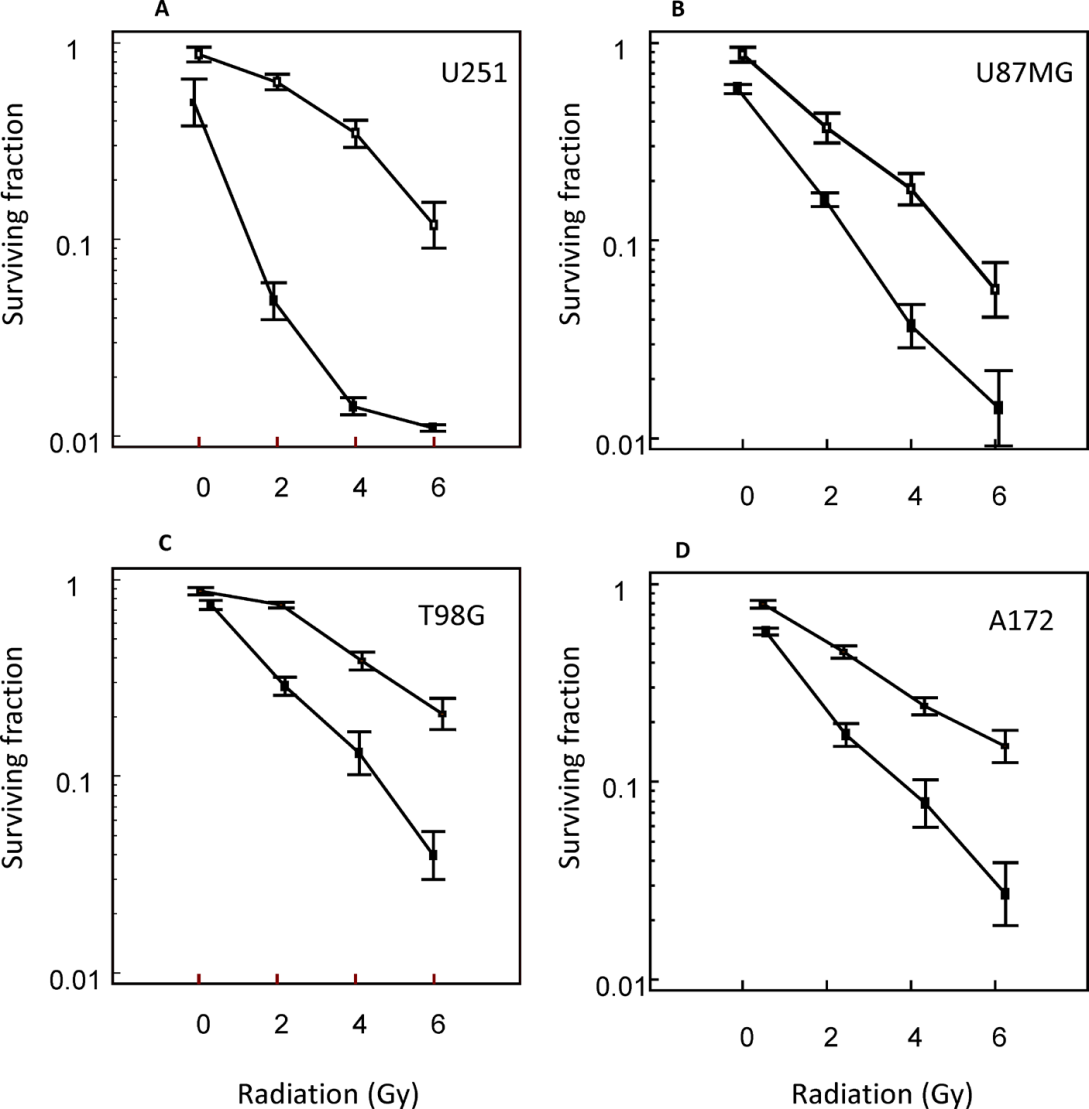
Radiosensitizing effects of vosaroxin on glioma cell lines Clonogenic curves for (**A**) U251, (**B**) U87MG, (**C**) T98G, and (**D**) A172 cells treated with vosaroxin (solid line) and control (no vosaroxin; dashed line).

The effects of vosaroxin on the radiosensitivity of glioma cells were assessed in clonogenic assays. Because drug exposure times were longer during clonogenic survival studies with radiation, vosaroxin concentrations equal to the IC_20_ in growth inhibition assays were used; IC_20_ values ranged between 10 nM and 100 nM. Treatment of glioma cells with vosaroxin alone resulted in a surviving fraction analyzed at 21 days of culture of 0.80 ± 0.064 in U251, 0.77 ± 0.18 in U87MG, 0.84 ± 0.036 in T98G, and 0.87 ± 0.18 in A172 cells, which is an appropriate degree of cytotoxicity for evaluation of vosaroxin in combination with radiation. In the combination protocol, 48 hours after drug exposure, cell cultures were irradiated at 2, 4, and 6 Gy and colony-forming efficiency was determined 21 days later in U251, U87MG, T98G, and A172 cells (Figure 3). This treatment resulted in a dose enhancement factor of 1.33 in U251, 1.55 in U87MG, 1.40 in T98G, and 1.24 in A172 cells, calculated at a surviving fraction of 0.10. Our results are in agreement with those of Gordon et al. 2012 [39].

### Assessments of the mechanism of radiosensitization

**Figure 4:**
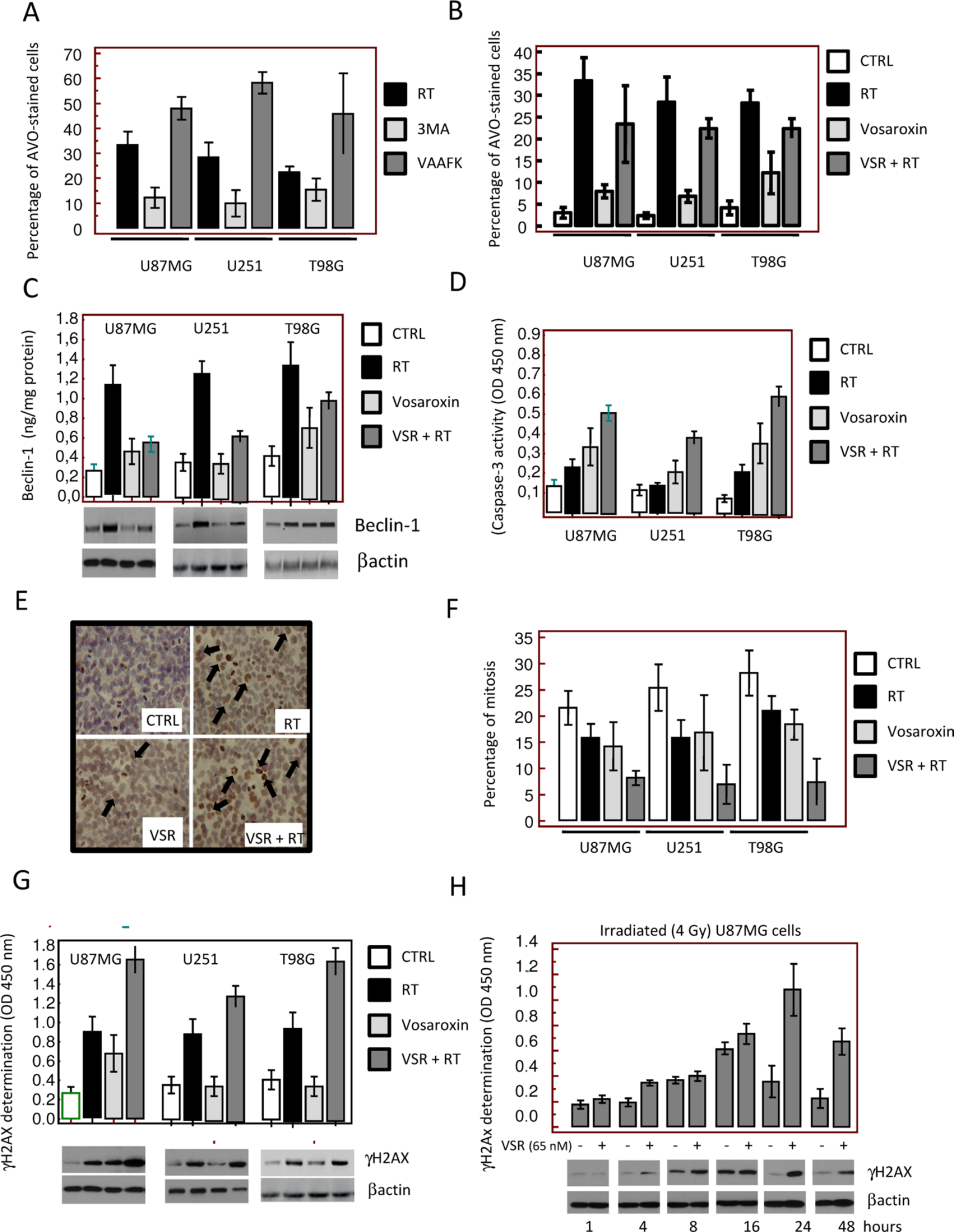
Molecular and cytologic analyses of the mechanisms of radiosensitization by vosaroxin. To define the molecular mechanisms involved in radiosensitization by vosaroxin we treated cells with radiotherapy (RT; at 2, 4, and 6 Gy) and vosaroxin at concentrations equal to IC_20_ values (15 nM for U251, 65 nM for U87MG, and 45 nM for T98G cells) (**A**) Percentage of acidic vesicular organelle (AVO)-stained glioma cells 24 hours after RT treatment (4 Gy). Percentage of AVO-stained cells was increased by the pan-caspase inhibitor z-Val-Ala-Asp(Ome)-fluoromethyl ketone (VAAFK, 10 μM) and was reduced by the autophagy inhibitor 3-methyladenine (3MA, 5 μM). (**B**) Percentage of AVO-stained cells after treatment with RT, vosaroxin (VSR), or the combination. CTRL: control. (**C**) Modulation of expression of an autophagy marker, beclin-1, by RT, vosaroxin, and combination vosaroxin plus RT by Western blot and ELISA. (**D**) Caspase-3 activity in treated and control cell cultures. (**E**) Immunocytochemical appearance of γH2Ax expression in T98G cultures after RT, vosaroxin, and combination RT plus vosaroxin for 24 hours. (**F**) Percentage of mitosis in U87MG, U251, and T98G cultures after single-agent or combination treatment. (**G**) γH2Ax expression in U87MG, U251, and T98G cells at 24 hours of treatment. (**H**) Expression of γH2Ax by ELISA and Western blotting measured in U87MG cells at 1, 4, 8, 16, 24, and 48 hours after 4 Gy irradiation with or without vosaroxin pre-treatment.

The mechanisms underlying neoplastic cell killing by ionizing radiation are largely unknown. We analyzed the modality of radiosensitization in U87MG, U251, and T98G cells as models of high, moderate, and low radiosensitivity, respectively. Increased autophagic responses have been associated with increased radioresistance [40, 41]; therefore, we analyzed cells treated with RT (4 Gy), vosaroxin (at concentrations corresponding to the IC_20_ value for each cell line), and the combination for the appearance of autophagy by evaluating the development of acidic vesicular organelles (AVOs) at 24 hours. RT increased the percentage of AVO-stained cells. As shown in Figure 4A, the levels of RT-induced AVO staining were further increased by the pan-caspase inhibitor z-Val-Ala-Asp(Ome)-fluoromethyl ketone (10 μM) and reduced by the autophagy inhibitor 3-methyladenine (5 μM). The addition of vosaroxin reduced AVO staining (Figure 4B), suggesting that vosaroxin inhibited RT-induced autophagy. Additionally, RT-induced levels of beclin-1, a marker for autophagy, were significantly reduced after vosaroxin treatment (Figure 4C), in agreement with AVO staining. In contrast, caspase-3 activity was increased with the addition of vosaroxin (Figure 4D), indicating potentiation of RT-induced apoptosis.

Since vosaroxin and RT can cause DNA double-strand breaks, levels of γH2Ax in treated cells were assessed by immunohistochemistry, Western blot, and enzyme-linked immunosorbent assay (ELISA). Increased γH2Ax expression following treatment with vosaroxin, RT, and the combination was observed in T98G cultures by immunohistochemistry (Figure 4E). The percentage of mitotic cells observed in these cultures was higher in control versus vosaroxin-treated cell cultures (Figure 4F); however, an increased presence of H2Ax-positive aberrant mitotic cells was observed after treatment with vosaroxin and/or RT (Figure 4E, black arrows). Expression of γH2Ax, as detected by Western blot and ELISA, increased after combination treatment (vosaroxin plus RT) compared with vosaroxin or RT alone in all cell lines tested (Figure 4G). In the absence of vosaroxin pretreatment, γH2Ax expression reached maximum levels at 16 hours after irradiation (4 Gy) and returned to baseline values after 24 hours (Figure 4H). In the presence of vosaroxin, the expression of γH2Ax was higher at each time point relative to non-pretreated cells; γH2Ax expression reached maximal levels at 24 hours and values did not return to baseline by 48 hours (Figure 4H). The increased γH2Ax levels observed with vosaroxin at later time points indicated a prolonged DNA damage response, possibly suggesting that double-stranded breaks were not sufficiently repaired in treated conditions.

### Vosaroxin increased the effects of radiotherapy in xenograft models of GBM

**Figure 5:**
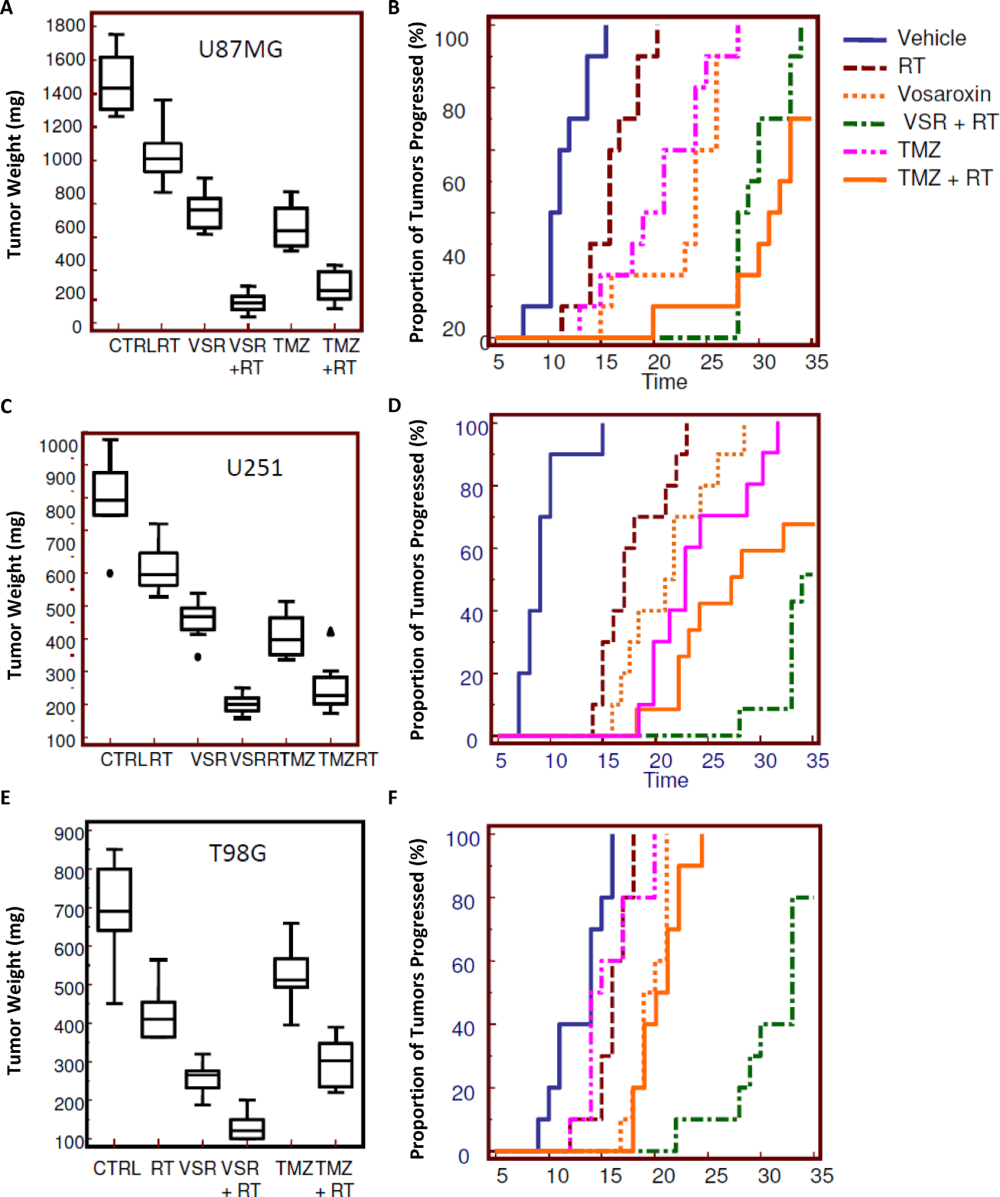
Radiosensitizing effects of vosaroxin on tumor weight and time to progression in xenograft models To assess the effect on tumors in an *in vivo* model, 1 × 10^6^ cells of U251, U87MG, and T98G GBM cells were subcutaneously injected in female cd1 nu/nu mice. When tumors reached a volume of 80 mm^3^ (about 10 days after cell injection), animals were randomized to receive radiotherapy (RT) alone (1 single dose of 4 Gy), vosaroxin (VSR; 10 mg/kg q 5 d for 5 wk), or vosaroxin (10 mg/kg q 5 d for 5 wk) plus RT (1 single dose of 4 Gy administered after 3 days of vosaroxin treatment). These treatments were compared with standard therapies consisting of temozolomide (TMZ; 16 mg/kg ′ 5 consecutive days) and temozolomide plus RT. Changes in tumor volumes were measured over time. After 35 days, animals were sacrificed and tumors harvested and weighed. Final tumor weights (at day 35) and Kaplan-Meier analysis of time to progression are shown for: (**A**, **B**) U87MG; (**C**, **D**) U251; and (**E**, **F**) T98G xenograft models. CTRL: control.

The effects of vosaroxin alone and in combination with RT were evaluated *in vivo* in U251, U87MG, and T98G GBM xenograft models. Effects on TTP and tumor weight after 35 days were compared to treatment with TMZ, as a single agent and in combination with RT (Figure 5).

In U87MG, U251, and T98G xenografts, final tumor weight was reduced by 44%, 42%, and 60%, respectively, with vosaroxin treatment compared with vehicle controls (Figure 5A, 5C, 5E). The addition of vosaroxin increased the antitumor effects of RT; combination treatment reduced final tumor weight by 87%, 79%, and 57% (compared with vehicle) whereas RT alone reduced final tumor weight by 30%, 23%, and 33% in U87MG (combination index [CI] = 0.52), U251 (CI = 0.48), and T98G (CI = 0.78) xenografts, respectively. The CI values suggested synergy between vosaroxin and RT in these tumor models. Temozolomide demonstrated efficacy similar to single-agent vosaroxin in U87MG and U251 cells, with final tumor weight reductions of 53% and 54% when compared with controls, while a smaller effect (25% reduction versus control) was observed in T98G cells. Temozolomide also increased RT sensitivity, with tumor weight reductions (compared with vehicle control) of 83% (CI = 0.85), 71% (CI = 0.74), and 57% (CI = 0.89), respectively. CI values for temozolomide were also in the range of synergism, but were higher than those observed with RT combined with vosaroxin.

Similar evidence of synergy was apparent when we assessed TTP (Figure 5B, 5D, 5F) in U87MG, U251, and T98G xenograft models. Hazard ratios comparing TTP with various treatments in U87MG, U251, and T98G xenograft models are shown in Table 2 (additional comparisons in Supplementary Table 1). In Kaplan-Meier analyses, probability of tumor progression in U87MG xenografts was reduced with RT or single-agent vosaroxin, compared with untreated animals (Figure 5B, Table 2). Vosaroxin in combination with RT significantly reduced the probability of tumor progression compared with RT or vosaroxin alone. In this radio- and chemosensitive model, temozolomide reduced tumor progression in comparison with untreated animals and was a good radiosensitizing agent in agreement with previously reported data, with no statistically significant differences in the radiosensitizing effects of vosaroxin versus temozolomide. Similar results were obtained for U251 xenografts (Figure 5D, Table 2). In this model, vosaroxin appeared to be a more effective radiosensitizer than temozolomide, although the difference was not statistically significant, likely due to small sample size. A more marked difference in the effects of vosaroxin and temozolomide on the probability of tumor progression was observed in the radio- and chemoresistant T98G xenograft model (Figure 5F, Table 2). In this model, vosaroxin demonstrated higher antitumor activity than temozolomide, in terms of tumor progression, both as single-agent treatment (*P* = 0.495) and in combination with RT (*P* < 0.0001).

**Table 2:** Probability of tumor progression in xenograft models

Treatments Compared	U87MG		U251		T98G	
	HR (95% CI)	*P* Value	HR (95% CI)	*P* Value	HR (95% CI)	*P* Value
VSR vs TMZ	0.6 (0.2–1.5)	0.20 (NS)	0.6 (0.3–1.6)	0.23 (NS)	0.5 (0.2–1.3)	0.0495
RT vs VSR + RT	6.2 (1.9–20.6)	< 0.0001	4.6 (1.5–14.8)	< 0.0001	4.6 (1.5–14.3)	< 0.0001
VSR vs VSR + RT	4.3 (1.2–15.6)	< 0.001	4.0 (1.3–12.1)	< 0.0001	4.7 (1.5–14.6)	< 0.0001
VSR vs TMZ + RT	0.8 (0.3–2.0)	0.64 (NS)	4.2 (1.4–12.5)	0.0002	1.0 (0.4–2.4)	0.97 (NS)
VSR + RT vs TMZ + RT	0.2 (0.1–0.8)	0.0054	0.5 (0.2–1.3)	0.11 (NS)	0.21 (0.1–0.7)	< 0.0001

CI: confidence interval; HR: hazard ratio for tumor progression; NS: not significant; RT: radiotherapy; TMZ: temozolomide; VSR: vosaroxin.

### Histopathological appearance of experimental gliomas

U87MG, U251, and T98G xenograft tumors grew rapidly with pleomorphism and high density of microvessels, which are typically seen in human GBM. Histopathological analysis revealed presence of glial neoplasia consisting of tightly packed sheets of heterogeneous tumor cell population with round to polygonal cell morphology (Supplementary Figure 1A), or spindle-shaped cell morphology with abundant, intensely eosinophilic cytoplasm and hyperchromatic nuclei and nucleoli (Supplementary Figure 1B). Bizarre gigantic cells with hyperchromatic round nuclei and elevated nuclear pleomorphism were also present. Tumor cells in rapid growth were dispersed on a fibrillar collagen background (Supplementary Figure 1C) that enveloped abundant vasculature (Supplementary Figure 1D). A narrow band of leukocyte infiltrate, consisting of granulocytes, B lymphocytes, and monocyte/macrophages, surrounded the tumors (Supplementary Figure 1E) and are indispensable components of the neoplastic microenvironment that can modulate the biological behavior of this malignancy. Tissues were characterized by pseudopalisading necrosis (present in central areas of tumors and in the largest tumors as uncontrolled tumor growth and subsequent hypoxia) in a garland-like arrangement of hypercellular tumor nuclei lining up around tumor necrosis-containing pyknotic nuclei (Supplementary Figure 1F). Additional features included thrombotic vessels and hemorrhage (Supplementary Figure 1G).

### Histopathological and immunohistochemical changes with RT and/or vosaroxin treatment

**Figure 6:**
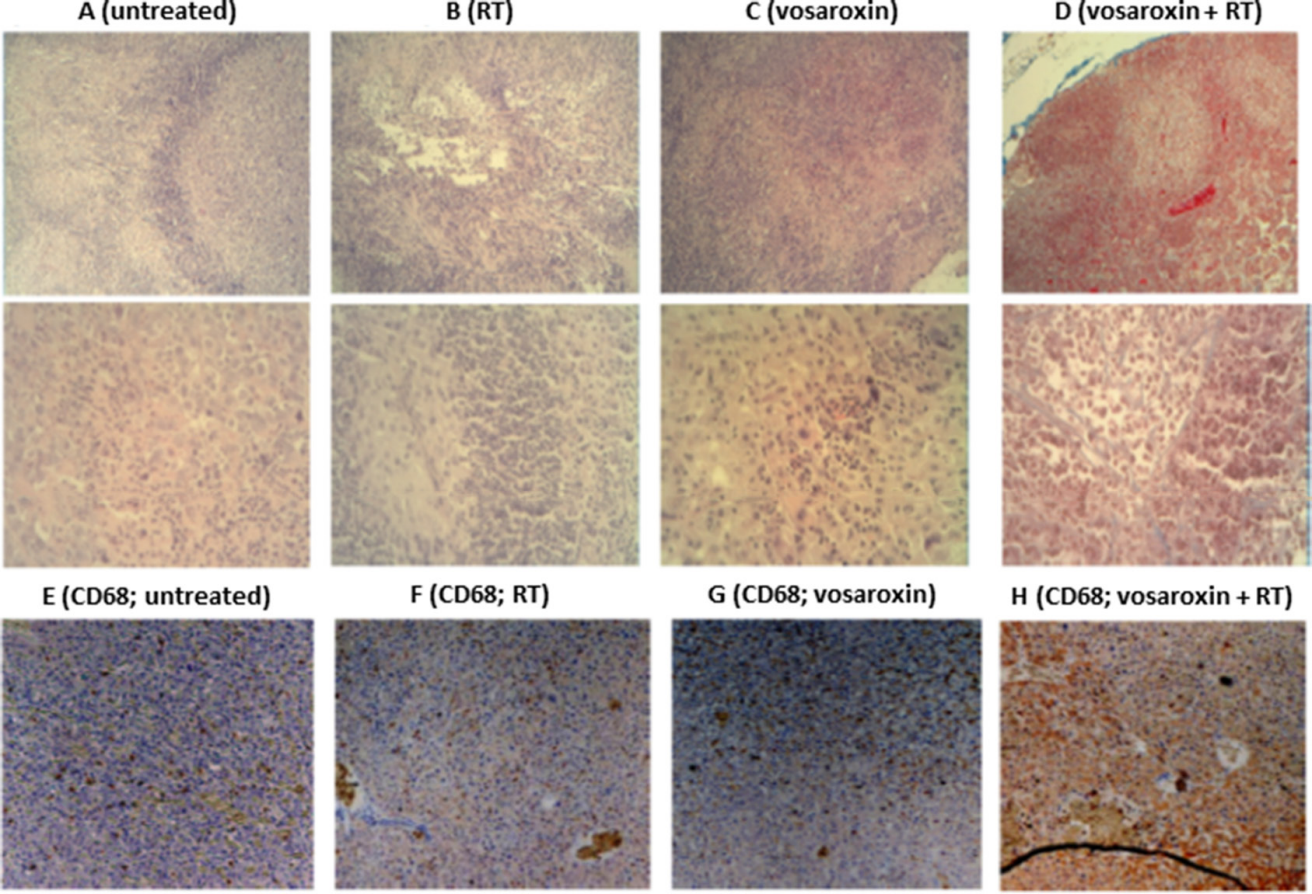
Leukocyte infiltration with vosaroxin and/or RT Panels (**A**–**D**) show the histologic appearance of U251 xenografts at low (50′; upper row) and high (400′; lower row) magnification in (A) untreated U251 xenografts, (B) after radiotherapy (RT), (C) after treatment with vosaroxin, and (D) after vosaroxin was added to RT. Panels (**E**–**H**) display immunostaining for CD68 expression at low magnification (50×) for untreated T98G tumors (E), and T98G tumors treated with RT (F), vosaroxin (G), and vosaroxin and RT (H).

Leukocyte infiltration was shown to be a common event in GBM growth, both in human patients and in experimental preclinical animal models (reviewed in Bienkowski and Preusser 2015 [42]). U251 xenografts demonstrated an infiltration of small rounded and mononucleated cells that were morphologically similar to leukocytes (Figure 6A). A smaller amount of granulocyte infiltration by multinucleated cells was also observed. Leukocytes were concentrated along the surface of the tumors but were also dispersed among the individual tumor cells. RT increased the accumulation/recruitment of leukocytes compared with untreated tumors and dense leukocyte infiltrates extended along the edges of growth (Figure 6B). A further increase in leukocyte recruitment was observed in tissues harvested from mice treated with vosaroxin (Figure 6C). When vosaroxin and RT were combined, the presence of leukocytes appeared to be associated with increased local proliferation of hematopoietic cells in dense clusters, suggesting a possible recruitment of circulating myeloid cells (Figure 6D). Staining with leukocyte-specific anti-CD68 antibodies suggested infiltration primarily by mononucleated monocytes, B lymphocytes, and natural killer cells (Figure 6E–6H).

Accumulating evidence supports the notion that RT triggers strong proimmunogenic effects with increased expression of both proinflammatory and anti-inflammatory/immune-tolerant cytokines produced by both tumor cells and murine stromal cells, including IL-1β, tumor necrosis factor (TNF)-α, TGF-β, IL-6, IL-8, and IL-10 (reviewed in Leroi et al. 2016 [43]). An important role of TNF-α has been its association with chemosensitivity in GBM. It has been shown that increased expression and secretion of biologically active TNF-α reduces P-glycoprotein expression and is associated with increased cytotoxicity of the MDR-relevant chemotherapeutic agents [44], which could result in a higher sensitivity to TMZ chemotherapy.

CD68 expression (a monocyte marker) was detected in treated T98G xenografts (Figure 6E–6H), and levels of pro- and anti-inflammatory cytokines were quantified from tissue extracts (Table 3). Cytokine levels were consistent with the morphological data and were suggestive of an active, acute inflammatory response following vosaroxin treatment. We observed that leukocyte recruitment and cytokine production were sustained after RT administration. Vosaroxin increased the production of proinflammatory (IL-1β, IL-6, and TNF-α) or angiogenetic (IL-8) cytokines, while the expression of stromal cell-derived factor (SDF)-1α, TGF-β1, and IL-10 was reduced with vosaroxin treatment (Table 3). The increased expression of SDF-1α and TGF-β1 agrees with previously reported data that suggested acute inflammation may switch from acute to chronic inflammation in response to increased tumor cell death after treatment. This switch may be associated with an adverse tumor microenvironment associated with a poor outcome [45, 46]. SDF-1α and TGF-β1 are involved in promoting tumor growth, whereas proinflammatory cytokines, such as Il-1β and TNF-α are mainly produced by classically activated (M1 polarized) tumor-associated microglia/macrophages. The latter display antitumor activity. Thus, the observed increase in proinflammatory cytokines may be an indication of an additional protective mechanism of vosaroxin (for review see Dello Russo C et al. 2016) [47].

**Table 3:** Cytokine expression in glioma xenograft models after treatment with radiation and/or vosaroxin

Cytokine	Treatment	U87MG	U251	T98G
IL-1β^a^	Control	60 ± 15	35 ± 7	21 ± 4
	RT	120 ± 40	77 ± 8	35 ± 6
	Vosaroxin	85 ± 10	44 ± 5	27 ± 4
	Vosaroxin + RT	425 ± 15	72 ± 13	124 ± 35
TNF-α^b^	Control	25 ± 12	5 ± 1	10 ± 1
	RT	43 ± 13	13 ± 3	18 ± 2
	Vosaroxin	17 ± 3	7 ± 1	3 ± 1
	Vosaroxin + RT	80 ± 12	44 ± 8	80 ± 5
IL-6^c^	Control	51 ± 7	120 ± 21	177 ± 15
	RT	60 ± 14	58 ± 12	72 ± 23
	Vosaroxin	78 ± 13	33 ± 8	30 ± 3
	Vosaroxin + RT	180 ± 44	684 ± 28	360 ± 40
IL-8^d^	Control	512 ± 28	213 ± 13	344 ± 44
	RT	750 ± 60	330 ± 44	535 ± 37
	Vosaroxin	715 ± 37	275 ± 35	440 ± 30
	Vosaroxin + RT	1200 ± 230	750 ± 48	917 ± 48
SDF-1α^e^	Control	11 ± 5	24 ± 6	18 ± 5
	RT	77 ± 12	43 ± 8	38 ± 6
	Vosaroxin	18 ± 3	32 ± 3	12 ± 4
	Vosaroxin + RT	57 ± 5	36 ± 5	22 ± 6
TGF-β1^f^	Control	43 ± 13	25 ± 5	35 ± 8
	RT	84 ±15	88 ± 12	75 ± 8
	Vosaroxin	47 ± 8	35 ± 6	27 ± 3
	Vosaroxin + RT	67 ± 12	62 ± 8	55 ± 7
IL-10^g^	Control	2.5 ± 0.3	< 1.0	< 1.0
	RT	34.2 ± 0.8	18.3 ± 0.5	35.2 ± 3.5
	Vosaroxin	6.2 ± 0.5	5.2 ± 0.6	< 1.0
	Vosaroxin + RT	12.6 ± 0.2	6.4 ± 0.5	5.2 ± 0.4

^a^Mouse IL-1β (pg/mg tissue). No human IL-1β was present.

^b^Human TNF-α (pg/mg tissue). 5–40 pg/mg tissue was also of mouse origin and was increased with treatments (data not shown).

^c^Human IL-6 (pg/mg tissue). Lower levels of IL-6 were observed when specific murine ELISA was used (about 10% of human IL-6).

^d^Human and mouse IL-8 (pg/mg tissue).

^e^Mouse SDF-1α. Contribution of human SDF-1α 5%–15%.

^f^Human TFG-β1.

^g^Mouse IL-10.

IL: interleukin; RT: radiotherapy; SDF: stromal cell-derived factor; TGF: transforming growth factor; TNF: tumor necrosis factor.

A previous study has suggested that CD38 and iNOS expression were associated primarily with M1 macrophages in the murine system, whereas Egr2, c-myc-1, and arginase-1 expression were exclusive associated with M2 macrophages [48]. To further define the role of TAMs in the antitumor effects of RT and vosaroxin, iNOS and arginase-1 expression were assessed in tissue extracts from U87MG, U251, and T98G xenografts following study treatments. The results showed low expression of M1-associated iNOS after RT, but with high expression levels of arginase-1 when compared with controls (Supplementary Figure 2). In contrast, vosaroxin treatment was associated with high expression of iNOS and low expression of arginase-1. When combined, the arginase-1 and iNOS expression levels were similar to those of vosaroxin alone.

**Figure 7:**
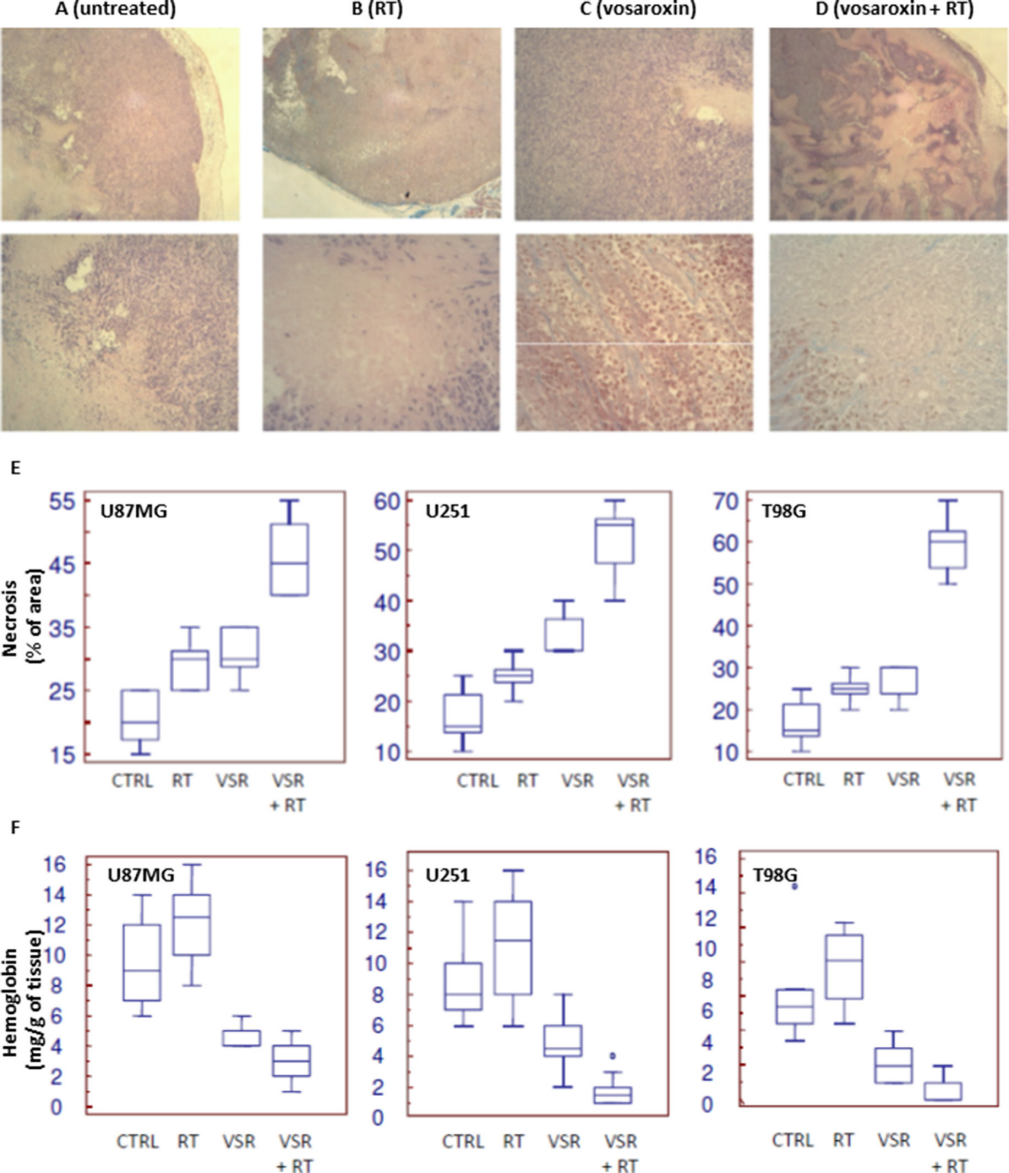
Necrosis in U251 xenograft tumors treated with vosaroxin and/or RT (**A**–**D**) staining for necrosis in U251 xenografts at low (50×; upper row) and high (400×; lower row) magnification in untreated tumors (A) and tumors treated with RT (B), vosaroxin (C), and RT plus vosaroxin (D). (**E**) Graphical analysis on percentage of necrotic cells in U87MG, U251, and T98G xenografts after various treatments. (**F**) Quantification of the amount of hemoglobin (as an indirect measure of vasculature) present in tissue extracts from U87MG, U251, and T98G xenografts after various treatments. CTRL: control; RT: radiotherapy; VSR: vosaroxin.

Necrosis in the xenograft tumor models was increased with treatment, representing 30%–40% of the tumor mass in the vosaroxin-treated animals and up to 70% in animals with U251 xenografts treated with vosaroxin and RT (Figure 7A–7D), and was increased in peripheral tumor zones. Quantification of necrosis is shown in Figure 7E. The increase in tumor necrosis is consistent with the observed elevation in leukocyte infiltration.

Tumor vasculature was also impacted by treatment. Tumors from control animals had more numerous and larger superficial blood vessels than treated animals. The impacted vessels occurred near the periphery of the tumor (near the tumor-host interface) and may display characteristics of tumor angiogenesis and angiogenesis associated with the wound response. In addition to considerable necrosis areas, hemorrhage and edema in the tissue surrounding the tumor growth region were observed in the normal parenchyma encircling the tumor; this appearance was increased after treatment with vosaroxin and vosaroxin plus RT. Tumors with necrotic regions have an inadequate blood supply and are expected to differ from well-vascularized tumors in response to treatment. The percentage of necrotic areas was significantly increased after combined treatment with vosaroxin and RT, suggesting a close correlation between study treatment and necrosis in all three models (Figure 7E). The quantification of hemoglobin in tumors (Figure 7F) revealed that treatments were able to reduce the influx of blood in tumor tissues in all 3 xenografts used for this analysis.

**Figure 8:**
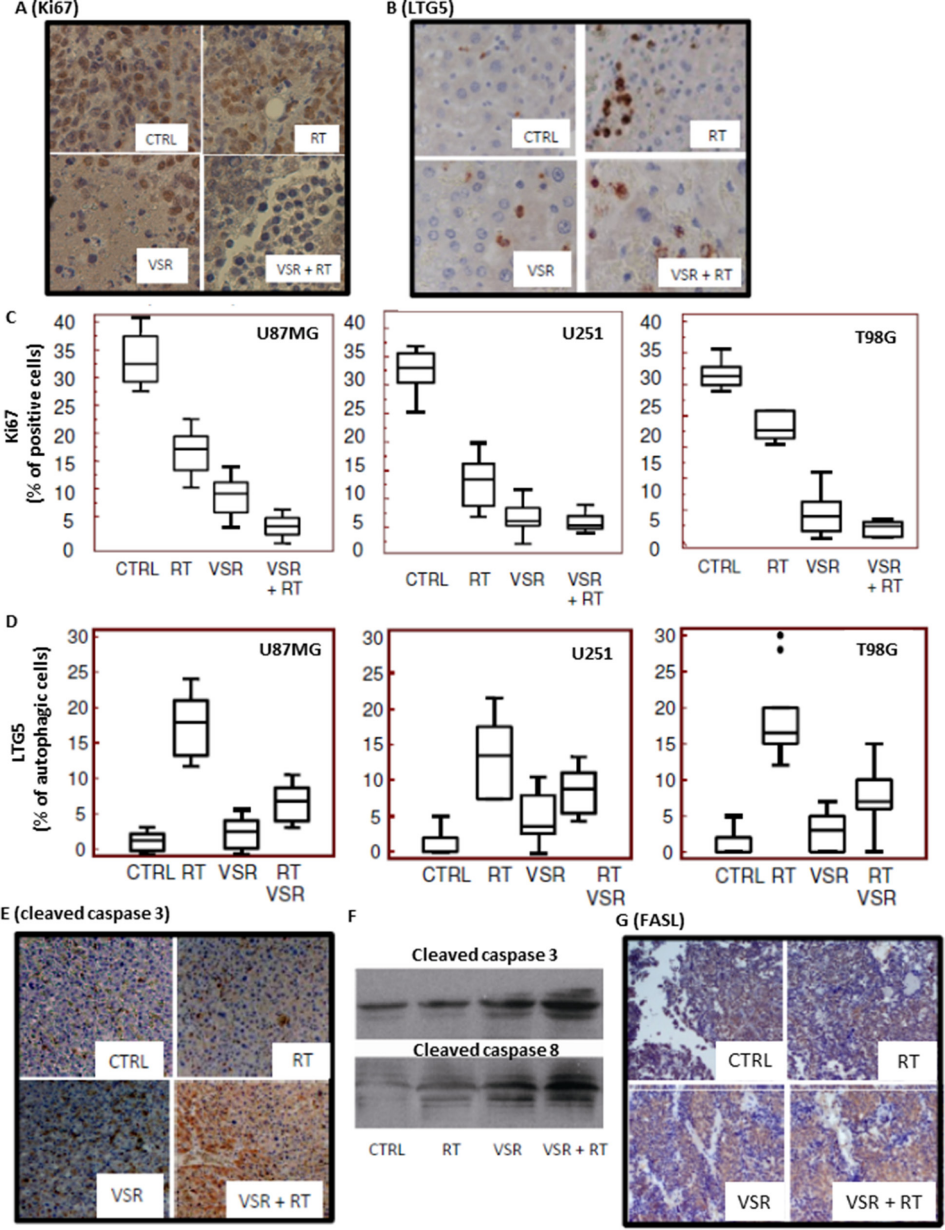
Expression of proliferation, autophagic, and apoptotic markers Immunohistochemical staining for expression of (**A**) Ki67 and (**B**) LTG5 in untreated and treated T98G xenografts. Quantification of Ki67 expression (**C**) and LTG5 expression (**D**) in U87MG, U251, and T98G xenografts. Immunohistochemical staining for caspase-3 expression (**E**) and Western blot analyses of caspase-3 and caspase-8 levels in tissue extracts (**F**) for untreated and treated T98G xenografts. (**G**) Immunohistochemical staining for FasL expression in the T98G xenograft model. CTRL: control; RT: radiotherapy; VSR: vosaroxin.

Increased fibrosis and reduced angiogenesis are related to reduced Ki67 expression. Ki67 expression was evaluated by immunohistochemistry in T98G xenografts treated with RT, vosaroxin, or the combination (Figure 8A). The percentage of Ki67-positive cells was markedly reduced with treatment (Figure 8C). Consistent with *in vitro* data, we also observed a reduction of LTG5, an autophagic molecular marker, with combination treatment relative to RT alone (Figure 8B and 8D). Massive apoptosis was associated with cleavage of caspase-3 and caspase-8 (Figure 8E, 8F) as well as increased FASL expression (Figure 8G) in T98G xenografts.

### Distribution of vosaroxin to brain in mice

After administration of [^14^C]-vosaroxin (20 mg/kg) to human nasopharyngeal tumor-bearing mice, radioactivity distributed rapidly, with only 2.6% to 1% remaining in the blood compartment at 8 hours postdose (Table 4). Maximum concentration in the cerebrum was 0.744 μgeq/g or 1.85 μMeq/g and in the cerebellum was 0.811 μgeq/g or 2.02 μMeq/g. Although brain tissues (cerebrum and cerebellum) showed tissue:plasma ratios ≤ 1.5, radioactivity in the brain achieved concentrations associated with anticancer activity *in vitro* [32, 39]. These data suggested that active levels of vosaroxin may be achieved in the brain clinically where dose levels similar to or higher than 60 mg/m^2^ have been investigated [37, 49, 50].

**Table 4:** Distribution of [^14^C]-vosaroxin to brain in human nasopharyngeal tumor-bearing mice

	Concentration μg equivalent of vosaroxin/mL or gram (tissue/plasma ratio)					
	5 min	30 min	1 h	3 h	8 h	24 h
**Plasma**	3.091 ± 0.104 (1.0)	1.885 ± 0.097 (1.0)	1.061 ± 0.071 (1.0)	0.544 ± 0.046 (1.0)	0.189 ± 0.006 (1.0)	< LOD
**Cerebrum**	0.573 ± 0.097 (0.2)	0.744 ± 0.056 (0.4)	0.559 ± 0.071 (0.5)	0.307 ± 0.007 (0.6)	0.275 ± 0.053 (1.5)	< LOD
**Cerebellum**	0.773 ± 0.081 (0.3)	0.811 ± 0.071 (0.4)	0.709 ± 0.055 (0.7)	0.607 ± 0.045 (1.1)	0.290 ± 0.042 (1.5)	< LOD

LOD: limit of detection.

### Orthotopic GBM model

The efficacy of vosaroxin was investigated in an orthotopic mouse model using luciferase-transfected U251 cells. We deliberately inoculated a small number of cells (3 × 10^3^) to simulate treatment postsurgery, where a low number of tumor cells remaining in the operatory bed were able to regrow into a recurrent lesion. Treatments were started 5 days after cell injection when no luciferase activity was detectable intracranially; the animals were treated for 35 days and followed for a maximum of 185 days.

**Figure 9:**
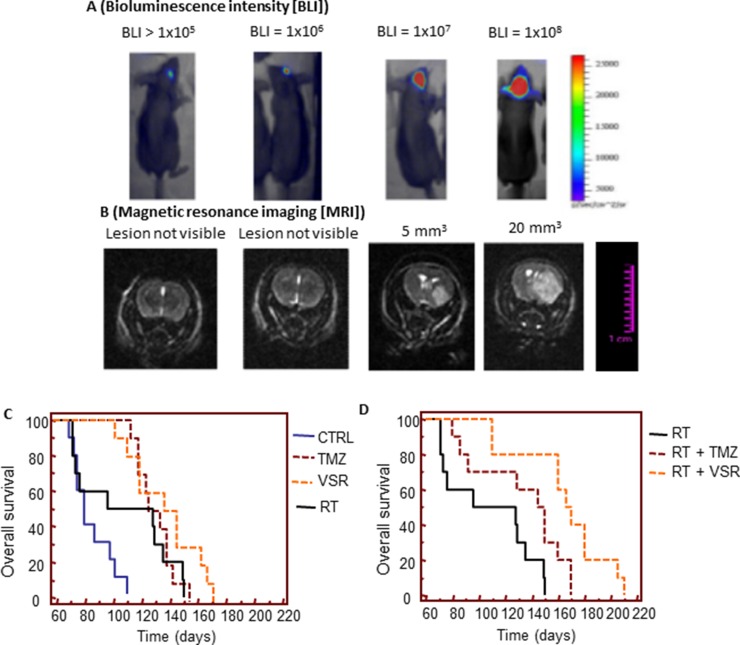
*In vivo* experiments: orthotopic intrabrain model (**A**) Representative images of relative bioluminescence intensity (BLI) in brain lesion recurrence after orthotopic cell injections. (**B**) Representative MRI images of brain lesion recurrence. (**C**) Comparison of the effects of single treatments versus control (CTRL) on overall survival. (**D**) Comparison of the radiosensitizing effects of temozolomide (TMZ) and vosaroxin (VSR) on overall survival. Note: images in 9A and 9B are not representation of dose- or time-response data.

Representative intrabrain lesions assessed by bioluminescence intensity and MRI are shown in Figure 9A and 9B. Control mice developed a bioluminescent lesion between 12 and 30 days with a mean of 18.1 ± 1.7 (standard error [SE]) days. Supplementary Figure 3A and 3B show recurrence probability over time (equivalent of disease-free survival [DFS]) and Figure 9C and 9D show overall survival (OS). Hazard ratios comparing DFS and OS with various treatments in U251 orthotopic models are shown in Table 5 (additional comparisons in Supplementary Table 2). RT increased DFS, slowing mean recurrence to 43.5 ± 2.9 days (*P* < 0.0001). Mean recurrence was also significantly slowed with vosaroxin (70.5 ± 7.3 days; *P* < 0.0001) and temozolomide (68.3 ± 5.4 days; *P* < 0.0001) treatment. Combination treatment further slowed mean time of recurrence: mean DFS with temozolomide plus RT was 82.5 ± 5.3 days (*P* < 0.0001 compared with temozolomide alone and *P* = 0.0023 compared with RT alone); mean DFS with vosaroxin plus RT was 96.5 ± 6.3 days (*P* = 0.0244 versus vosaroxin alone and *P* = 0.0088 versus temozolomide plus RT). These data indicated that vosaroxin produced greater increases in DFS than temozolomide, alone and in combination with RT, in MGMT-negative glioblastomas. Similar results were observed in analyses of OS in mice treated with vosaroxin or temozolomide alone or in combination with RT.

**Table 5:** Hazard ratios for disease-free survival and overall survival in orthotopic U251 models

Treatments Compared	Disease-free Survival		Overall Survival	
	HR (95% CI)	*P* Value	HR (95% CI)	*P* Value
TMZ vs VSR	2.0 (0.9–5.5)	0.06 (NS)	3.0 (1.0–8.8)	0.0446
RT vs VSR + RT	3.5 (1.2–9.9)	0.0007	9.9 (2.9–34.1)	< 0.0001
VSR vs VSR + RT	2.4 (1.1–8.8)	0.0244	4.5 (2.2–22.5)	0.0001
VSR vs TMZ + RT	1.2 (0.4–3.2)	0.66 (NS)	1.5 (0.5–3.0)	0.78 (NS)
VSR + RT vs TMZ + RT	2.5 (1.0–6.2)	0.0088	3.7 (1.2–11.0)	0.0198

CI: confidence interval; HR: hazard ratio; NS: not significant; RT: radiotherapy; TMZ: temozolomide; VSR: vosaroxin.

## DISCUSSION

The potential of topoisomerase II as a target for radiosensitization has been previously suggested in studies with other agents in experimental tumor models [20–23, 39, 51]. Radiation causes cells to arrest in G2/M phase, which is when topoisomerase II typically functions in replication and repair. Topoisomerase II inhibitors can cause G2 arrest, which places cells in a relatively radiosensitive phase of the cell cycle [39, 51, 52]. However, the clinical utility of topoisomerase II inhibitors is limited by systemic toxicity and drug resistance that is frequently mediated by P-glycoprotein. [18, 19, 24, 25].

Vosaroxin is a novel topoisomerase II inhibitor that is not a substrate for P-glycoprotein drug pumps, and can induce apoptosis independent of p53, thereby avoiding two common mechanisms of drug resistance [29]. It has been shown to be active against various preclinical models and demonstrated synergistic activity in combination with other antineoplastic agents [29–35].

In this study, we evaluated vosaroxin in preclinical models of GBM with and without RT and showed that vosaroxin’s antitumor effects in GBM models were not impacted by MGMT, p53, or PTEN expression.

The data presented here indicate that there was no significant initial increase in DNA damage based on γH2Ax expression at early time points after radiation, suggesting no increase in the number of DNA double-strand breaks. However, at 24 hours after radiation, there were increased γH2Ax foci, suggesting that vosaroxin inhibits the repair of radiation-induced DNA damage in GBM cells. A previous study evaluating the topoisomerase II inhibitors amrubicin and amrubicinol in lung adenocarcinoma showed enhancement of radiosensitivity similar to the results reported here [20–23, 39]. Similar to our findings, this study showed increased necrosis when cells were irradiated and treated with topoisomerase inhibitors. In our study, we also demonstrated an increased proportion of necrotic cells *in vivo* after radiation and vosaroxin treatment, with subadditive increases in percent necrosis in the combination-treated cells. Vosaroxin was able to cross the blood-brain barrier and infiltrate brain tumors at concentrations compatible with IC_50_ values identified in this study.

We hypothesize that vosaroxin may induce radiosensitization through an enhancement of apoptosis and reduced autophagy. It has been recently demonstrated that topoisomerase IIα is highly expressed in glioblastoma stem cell lines and that inhibition of topoisomerase IIα with siRNA decreases cell proliferation and induces apoptosis in germline stem cells (GSCs) [53]. This effect may amplify the antitumor effects of vosaroxin through reduction of tumor growth and tumor recurrence by GSCs. The effect on GSCs may possibly be increased by reducing the expression of pro-stem cell regrowth factors (inflammatory cytokines TGF-β1, IL-10, and SDF-1α).

As this is the first study to evaluate the radiation-sensitizing properties of vosaroxin, we further explored possible mechanisms of these properties. Increasing evidence suggests that an inflammatory, immunosuppressive tumor microenvironment may promote invasion by GBM cells [54, 55] through the activation of pathways that recruit myeloid precursors. One interesting possible mechanism involved in the sensitivity of GBM tumors to vosaroxin and vosaroxin plus RT is the recruitment of myeloid cells such as monocytes, macrophages, and microglia. This process plays a crucial role in neuroinflammation and has been recently identified as a novel therapeutic target, especially for chronic forms of neuroinflammation [47].

Macrophage functions are generally categorized as M1 or M2. M1 refers to the classically activated, polarized macrophages, while M2 refers to the alternatively activated macrophages. M1 macrophages differ from M2 in terms of receptor expression, cytokine production, effector functions, and chemokines. M1 macrophages are differentiated by microbial products such as LPS, by IFNγ-produced TH1 cells during an adaptive immune response, or by natural killer cells during an innate immune response. M1 macrophages have tumor-killing capacity and express a number of factors including iNOS, IL-1β, and TNF-α. In contrast, M2 macrophages differentiate to several subtypes dependent on external stimulation [47].

The preliminary characterization of M1 and M2 macrophages in this study suggested that vosaroxin induces an acute response from M1 macrophages, while RT is associated with M2 macrophage activity and may be associated with an elevated risk of recurrence. However, additional molecular characterization is necessary to obtain conclusive data and will be the subject of a separate report.

The possible involvement of proliferating monocytes is also invoked due to the presence of close “germinal cores/cluster” dispersed in the necrotic tumor masses. This event could have dual effects: to participate in the elimination of necrotic cells (resolution) or to mediate the awakening of quiescent stem cells (leading to recurrence). The latter effect was not supported by results in the orthotopic models, which demonstrated no increase in recurrence with vosaroxin and the vosaroxin plus RT combination after only 35 days of treatment (1 treatment cycle). The rate of recurrence and the survival percentage in combination treatment were significantly better than those observed for the standard treatment, temozolomide plus RT, providing evidence against recurrence due to stimulation of cancer stem cells.

These data demonstrate that vosaroxin is a broadly active antitumor agent *in vitro* and *in vivo*, with potent activity in aggressive and temozolomide-resistant glioblastoma tumor models, supporting ongoing clinical evaluation of this compound both alone and in combination with RT for the treatment of postsurgery glioblastoma patients. A limitation of this study is that no direct comparison of the antitumor effects between vosaroxin and other topoisomerase inhibitors was performed. This topic may be of interest to others in future research.

## MATERIALS AND METHODS

### Reagent and drug preparation

All the materials for tissue culture were purchased from HyClone (Cramlington, UK). Plasticware was obtained from Nunc (Roskilde, Denmark). Antibodies for β-actin (sc-130065), p-DNA-PKCs (Thr 2609; sc-101664), Rad-51 (sc-8349), γ-H2AX (Ser 139; sc-101696), FAS (C-20; sc-715) and FAS-L (N-20; sc-834], CD68 (H-255; sc-9139), CD20 (M20; sc-7735), matrix metalloproteinase (MMP)-2 (4D3; sc-53630), and CXCR4 (4G10; sc-53534) were purchased from Santa Cruz Biotechnology (Santa Cruz, CA, USA). Survivin antibody was purchased from Biorbyt (Cambridge, UK). Vosaroxin was kindly provided by Sunesis Pharmaceuticals, Inc. For *in vitro* cell viability assays, vosaroxin was dissolved in 0.17% methanesulfonic acid (Sigma-Aldrich Chemical Co., St. Louis, MO, USA), forming a stock solution of 10 mM. Working solutions were made by dilution of the stock solution with cell culture media. The *in vivo* formulation of vosaroxin (10 mg/mL) was used for *in vivo* studies and further diluted for injection into mice by dilution with vehicle (0.17% methanesulfonic acid in 5% sorbitol; formulation reagents from Sigma-Aldrich). Temodal^®^ (temozolomide) was purchased from Selleckchem labs (Aurogene, Rome, Italy). Anti-MIB1 (Ki67) was purchased from Dako (Dako Italia SPA, Milan, Italy). Pan-caspase inhibitor z-Val-Ala-Asp(Ome)-fluoromethyl ketone and autophagy inhibitor 3-methyladenine were purchased from Sigma-Aldrich.

### Cell lines

Thirteen human glioma cell lines (U251, U373, SNB19, U118, U138, U87MG, A172, LN229, LN19, SW1783, T98G, SF-268, and D54) were cultured at 37°C in 5% CO_2_ and were maintained in Dulbecco’s modified Eagle medium (DMEM) containing 10% (v/v) fetal bovine serum, 4 mM glutamine, 100 IU/mL penicillin, 100 μg/mL streptomycin, and 1% nonessential amino acid (Invitrogen Life Technologies, Inc., Rockville, MD, USA). To minimize the risk of working with misidentified and/or contaminated cell lines, the cells used in studies reported here were stocked at very low passages after initial receipt from the vendor to reduce the possibility of contaminated cell line stocks and used at < 20 subcultures. Periodically, DNA profiling by GenePrint^®^ 10 System (Promega Corporation, Madison, WI, USA) was carried out to authenticate cell cultures. Luciferase-transfected U251 cells were kindly provided by Jari E. Heikkila, department of Biochemistry and Pharmacy, Abo Akademi University, Turku, Finland. Three GBM patient-derived stem cell lines, BT12M, kindly provided by J. Gregory Cairncross and Samuel Weiss (Hotchkiss Brain Institute, Faculty of Medicine, University of Calgary, Calgary, Alberta, Canada) [56] and CSCs-5 and CSCs-7 from Marta Izquierdo (Departamento de Biología Molecular, Universidad Autónoma de Madrid, Spain) [57], were maintained as neurosphere cultures in Neurocult medium (Stem Cell Technologies, Vancouver, BC, Canada) supplemented with epidermal growth factor (20 ng/mL) and fibroblast growth factor (10 ng/mL). These non-commercially available, patient-derived cells were analyzed using short tandem repeat profiles. The expression of MGMT, p53, and PTEN was detected in western blots with antibodies from Santa Cruz against MGMT (sc166528), wt p53 (sc-100), total p53 (sc-126), and PTEN (sc-7974).

### Inhibition of proliferation assays

Cells were seeded at a density of 2 × 10^4^ cells/mL in 24-well plates. Cells were center to attach and grow in 5% fetal bovine serum in DMEM for 24 hours. Cells were treated with different doses of radiation or temozolomide and then maintained in the appropriate culture conditions. At the conclusion of the treatment period, cells were trypsinized and resuspended in 1.0 mL of saline; viable cells were counted using the NucleoCounter^™^ NC-100 (Chemotec, Cydevang, Denmark). All experiments were conducted in triplicate. IC_50_ values were calculated using GraFit (Erithacus Software Limited, Staines, UK).

### Cell cycle and apoptosis analyses

Cell cycle analyses were performed using FxCycle™ Far Red (Life Technologies Europe BV, Monza, Italy). Apoptosis was analyzed with the Alexa Fluor^®^ 488 Annexin V/Dead Cell Apoptosis Kit. Both analyses were measured on a Tali® image-based cytometer quantifying fluorescence emission at 530 nm (eg, FL1) and > 575 nm [58]. The results were expressed as the ratio of apoptosis in treated cells to apoptosis in vehicle-treated control cells.

### Clonogenic survival assays

For clonogenic survival assays, exponentially growing cells (70% confluence) were cultured in regular media and treated with vosaroxin, at the appropriate concentrations, or vehicle (final dimethyl sulfoxide concentration of 0.1%) for 48 hours. Tumor cells were then irradiated at room temperature with increasing doses of radiation (0–6 Gy) using an x-ray linear accelerator (dose rate of 200 cGy/min). Nonirradiated controls were handled identically to the irradiated cells with the exception of the radiation exposure. After treatment, cells were diluted at the appropriate concentration (1000 cells in 10 mL) and reseeded into a new 100 mm tissue culture dish (in triplicate) and incubated for 14 days. At day 14, the medium was removed and colonies were fixed with methanol:acetic acid (10:1, v/v), and stained with crystal violet. Colonies containing more than 50 cells were counted. The plating efficiency was calculated as the number of colonies observed divided by the number of cells plated. The surviving fraction was calculated as the number of colonies formed in the treated dishes compared with the number formed in the control. The survival curves were analyzed using SPSS statistical software (Chicago, IL, USA) by means of a fit of the data by a weighted, stratified, linear regression, according to the linear-quadratic formula: S(D)/S(O) = exp-(αD + βD2). The cell survival enhancement ratio was calculated as the ratio of the mean inactivation dose under control conditions divided by the mean inactivation dose after treatment as previously described [59, 60].

### Detection and quantification of autophagic cells by staining with acridine orange

As a marker of autophagy we evaluated the presence of AVOs after staining of cell cultures with LysoTracker^®^ Red DND-99 kit (Life Technologies Italia, Monza, Italy) according to the manufacturer’s instructions. The intensity of the red fluorescence is proportional to the degree of acidity. The percentage of autophagic cells was quantified with the Tali^®^ Image-Based Cytometer (Life Technologies) measuring fluorescence emission at 590 nm with fluorescence excitation at 577 nm. The results were expressed as the percentage of cell AVOs stained in controls and in treated cultures.

### Preparation of cell lysates and Western blot analysis

**C**ells grown in 90 mm diameter Petri dishes were treated and at various timepoints were washed with cold phosphate-buffered saline (PBS) and immediately lysed with 1 mL radioimmunoprecipitation assay (RIPA) buffer containing protease and phosphatase inhibitor cocktail. Total lysates were electrophoresed in SDS-PAGE (7%), and separated proteins transferred to nitrocellulose and probed with the appropriate antibodies using the conditions recommended by the suppliers. Protein levels in total extracts were normalized to actin.

### ELISA determinations

After appropriate treatments, tumor cell cultures and tissues were harvested for analysis of cytokine expression. Cell pellets were washed with PBS and lysed with RIPA buffer. Cell lysates and conditioned media were assayed by ELISA to measure (i) active human caspase-3 (CBA045, Merck Chemicals Ltd., Nottingham, UK), (ii) beclin-1 (E98557Hu, USCN Life Sciences, Houston, TX, USA), and (iii) DNA damage (EpiQuik *in situ* DNA Damage Assay Kit, Epigentek, Farmingdale, NY, USA). Tumor extracts were analyzed for the presence of:

- MMP-2 (KHC3081)
- TNF-α (KHC3011, Life Technologies Italia, Monza, Italy)
- CXCR4 (Cyto Glow CXCR4 [pSer339], cell-based ELISA, Assay Biotech, Sunnyvale, CA, USA)
- Human Fas ligand (ELH-FASL, RayBiotech, Norcross, GA, USA)
- IL-6 (orb50052) and survivin (orb50135) (Biorbyt)
- TGF-β1 (Quantikine ELISA Kit, DB100B, R&D Systems, Minneapolis, MN, USA)
- SDF-1α (Human CXCL12/SDF-1α Quantikine ELISA Kit, DSA00, R&D Systems)
- Mouse IL-10 DuoSet ELISA DY417 (R&D Systems Minneapolis, MN, USA)
- Mouse NOS2 / iNOS ELISA Kit (Sandwich ELISA) - LS-F12166 (LifeSpan Biosciences, Inc, Seattle, WA, USA)
- Mouse ARG1 / Arginase-1 ELISA Kit (Sandwich ELISA) - LS-F10883 (LifeSpan Biosciences, Inc, Seattle, WA, USA)

All determinations were performed in triplicate, according to manufacturers’ instructions. Data are presented as mean ± SE. Cytokine secretion determined by ELISA was normalized to total protein concentration in tissue lysates.

### Mouse glioblastoma xenograft model

Female CD1-nu/nu mice, at 6 weeks of age, were purchased from Charles River (Milan, Italy) under the guidelines established by our institution (University of L’Aquila, Medical School and Science and Technology School Board Regulations, complying with Italian government regulation n.116, January 27, 1992, for the use of laboratory animals). All mice received subcutaneous flank injections (2 each) of 1 × 10^6^ U251, U87MG, and T98G cells representing models for MGMT-negative and MGMT-positive cells. Tumor growth was assessed biweekly by measuring tumor diameters with a Vernier caliper (length × width). Tumor weight was calculated according to the formula tumor weight (mg) = tumor volume (mm^3^) = d2 × D/2, where d and D are the shortest and longest diameters, respectively. The effects of the treatments were assessed as previously described [36]. At about 10 days after the tumor injection, 30 mice with tumor volumes of 0.5–0.8 cm^3^ were randomly divided into 6 groups (5 mice per group with 2 tumors each): (1) control (vehicle); (2) vosaroxin (10 mg/kg every 5 days for 5 weeks intravenous [IV] injection into tail vein; (3) radiotherapy (RT; 4 Gy delivered in a single fraction) [59]; (4) vosaroxin in combination with RT; (5) temozolomide (16 mg/kg 5 consecutive days) and (6) temozolomide in combination with RT. Anesthetized tumor-bearing mice received focal irradiation. Irradiation was delivered using an x-ray linear accelerator at a dose rate of 200 cGy/min at room temperature. All mice were shielded with a specially designed lead apparatus to allow irradiation to the right hind limb. At the end of the study (35 days after the start of treatments), animals were sacrificed by carbon dioxide inhalation and tumors were then removed surgically. Half of the tumor was directly frozen in liquid nitrogen for protein analysis and the other half was fixed in paraformaldehyde overnight for immunohistochemical analyses.

### Immunohistochemical evaluations

Indirect immunoperoxidase staining of tumor xenograft samples was performed on paraffin-embedded tissue sections (4 μm); sections were incubated with primary antibodies overnight at 4°C. Avidin-biotin assays were performed using the Vectastain Elite kit (Vector Laboratories, Burlingame, CA, USA). Mayer’s hematoxylin was used as nuclear counterstain. Tumor microvessels were counted at 400× magnification in 5 arbitrarily selected fields and the data were presented as number of CD31-positive microvessels/100× microscopic field for each group. Ki67 labeling index was determined by counting 500 cells at 100× and determining the percentage of cells staining positively for Ki67. Apoptosis was measured as the percentage of tunnel positive cells ± standard deviation (SD) measured on 5 random fields (400×). The presence of hematopoietic cells in tumor tissue and in blood vessels as well as the presence of microthrombi and bleeding zones was demonstrated by Martius yellow-brilliant crystal scarlet blue technique. Tumor hemoglobin levels were also quantified as previously described [61].

### Evaluation of treatment response *in vivo* (xenograft model)

The following parameters were used to quantify the antitumor effects of different treatments as previously described [59]: tumor volume, measured throughout the experiment; tumor weight, measured at the end of the experiment; tumor progression, defined as an increase of greater than 50% of tumor volume with respect to baseline; and time to progression (TTP), defined as the time until tumor progression was observed from baseline. Tumor size was measured with calipers and the TTP of a single tumor was calculated. TTP data were used to generate Kaplan-Meier curves for progression. This method of analysis was described previously [59, 62], and may reduce both inter-subject variability resulting from differences in engraftment efficacy as well as intra-subject variability in response.

*In vivo* drug combination studies were evaluated using CalcuSyn. For CI calculations of dual administration, the values of % cell kill (%CK) for a fixed tumor volume were determined by the log CK (LCK). LCK was calculated as LCK = (T − C) / (3.32 × Td), where Td represented the mean control group doubling time required to reach a fixed tumor volume, expressed in days. T and C were the mean times in days required to reach the same fixed tumor volume in the treated group and the control group, respectively. CK was calculated as %CK = [1 − (1 − 10LCK)] × 100. Tumor growth delay (TGD) was calculated as %TGD = [(T − C) / C] × 100, where T and C were the same values as those reported by Bruzzese et al. [63].

### Distribution of [^14^C]-vosaroxin to brain

A single IV dose of [^14^C]-vosaroxin (1.84 MBq/kg with 20 mg/kg unlabeled vosaroxin) was administered to nude mice bearing human nasopharyngeal tumors. Radiochemical purity was > 96%. For determination of plasma and brain concentrations, 3 mice/time point were sacrificed at 5 minutes, 30 minutes, and 1, 3, 8, 24, and 96 hours postdose. Blood samples were collected for plasma, and the cerebrum and cerebellum were harvested following exsanguination. Radioactivity in plasma was counted directly after addition of tissue-dissolving solution and scintillation liquid. Radioactivity in brain was determined after combustion. Radioactivity concentrations were converted into vosaroxin concentrations and expressed as mean ± standard error of the mean. When the statistical error of the counted sample radioactivity exceeded 5% of the counts, the sample radioactivity was defined to be under the reliable limit of measurement.

### Orthotopic intra-brain model

Orthotopic luciferase-transfected U251 tumor growth and therapy studies were conducted with an approved animal-use protocol. Nude mice were inoculated intracerebrally [64] as follows: animals were anesthetized with 100 mg/kg ketamine, 15 mg/kg xylazine, and 0.05 mL atropine intramuscularly; the surgical zone was swabbed with Betadine solution and the eyes coated with Lacri-lube; the head was fixed in a stereotactic frame (mouse stereotaxics instrument, Stoelting Europe, Dublin, Ireland) and a midline scalp incision was made; a small hole was made at 1.0 mm anterior and 2 mm lateral to the exposed bregma; a sterile 5 μL Hamilton syringe with a 26-gauge needle was inserted at a depth of 3.0 mm from the skull surface and withdrawn by 0.5 mm to inject 3 × 10^3^ U87MG cells in a volume of 3 μL at an injection up to 1 μL/min. After the implantation of the tumor cells, the needle was center in place for 5 minutes to prevent reflux. The needle was then completely withdrawn from the brain over the course of 4 minutes (1.0 mm/min) and the skin was sutured. Just before treatment initiation (5 days after injection), animals were randomized to treatment groups of 10 mice each. *In vivo* bioluminescence images were obtained using a Hamamatsu camera (Hamamatsu Photonics Italy S.R.L, Arese, Italy) to identify intracranial implants similar to the method described by Kemper et al. [65]. Animals were anesthetized and luciferin (150 mg/kg) was injected intraperitoneally 15 minutes prior to imaging. The mice were photographed while placed on their front and the bioluminescence intensity (BLI) was measured in the region of interest. The BLI value just prior to the initiation of the treatment was used to calculate the %BLI of increment for each individual animal. We deliberately inoculated a small number of cells (3 × 10^3^) to simulate a chemo-radiotherapeutic treatment made after surgery in which a low number of tumor cells, remaining in the operatory bed, regrows and gives rise to a recurrence as described previously [62]. Treatments were started 5 days after cell injection when no luciferase activity was intracranially detectable. Tumor growth delay (recurrence time equivalent of DFS) was evaluated by assessment of intracranial luciferase activity by BLI, and also by magnetic resonance imaging (MRI) [66]. BLI photon counts and tumor volumes from MRI were correlated. Mice were euthanized when they displayed neurological signs (eg, altered gait, tremors/seizures, lethargy) or weight loss of 20% or greater of presurgical weight and OS was recorded.

### Magnetic resonance imaging

All MRI studies were performed using a MR750w 3.0T scanner (GE Healthcare, Little Chalfont, UK) with a 16-channel phased array flex wrist coil. Mice were placed prone in a plastic holder for ease of fixation and anesthetized to avoid movement during imaging. All images were obtained in the transverse plane using the following sequences: transverse T2-weighted turbo spin-echo sequence (repetition time msec/echo time msec) 6766/120, echo train length of 25, one signal acquired, matrix of 192 × 192) applied with a section thickness of 0.9 mm, an intersection gap of 0.0 mm, and a flip angle of 160°. The field of view was 36 × 60 mm^2^, which included the tumor in its entirety (20 sections) with a resultant voxel size of 0.3 × 0.3 × 1.0 mm^3^.

### Statistics

Continuous variables were summarized as mean and SD or as median and 95% confidence interval. For continuous variables not normally distributed, statistical comparisons between control and treated groups were established by Kruskal-Wallis test. When the Kruskal-Wallis test revealed a statistical difference, pairwise comparisons were made by the Dwass-Steel-Critchlow-Fligner method and the probability of each presumed “non-difference” was indicated. For continuous variables normally distributed, statistical comparisons between control and treated groups were established by analysis of variance (ANOVA) test or by Student *t*-test for unpaired data (for 2 comparisons). When the ANOVA test revealed a statistical difference, pairwise comparisons were made by Tukey’s honestly significant difference test and the probability of each presumed “non-difference” was indicated. Dichotomous variables were summarized by absolute and/or relative frequencies. For dichotomous variables, statistical comparisons between control and treated groups were established by the Fisher’s exact test. For multiple comparisons, the level of significance was corrected by multiplying the *P* value by the number of comparisons performed (n) according to Bonferroni correction. TTP was analyzed by Kaplan-Meier curves and Gehan’s generalized Wilcoxon test. When more than 2 survival curves were compared, the log-rank test for trend was used to test the probability of a trend in survival scores across the groups. All tests were 2-sided and were determined by Monte Carlo significance. *P* values < 0.05 were considered statistically significant. SPSS statistical analysis software package version 10.0 and StatDirect version 2.3.3 (StatsDirect Ltd, Altrincham, UK) were used for statistical analysis and graphic presentation.

## SUPPLEMENTARY MATERIALS FIGURES AND TABLES



## Abbreviations

ANOVA: analysis of variance
AVO: acidic vesicular organelles
BLI: bioluminescence intensity
CI: combination index
DFS: disease-free survival
DMEM: Dulbecco’s modified Eagle medium
ELISA: enzyme-linked immunosorbent assay (defined at second use
GBM: glioblastoma multiforme
GSC: germline stem cell
IC_50_: 50% inhibitory concentration
IL: interleukin
IV: intravenous
MDR: multidrug resistance
MGMT: O6-methylguanine methyltransferase
MMP: matrix metalloproteinase
MRI: magnetic resonance imaging
OS: overall survival
PBS: phosphate-buffered saline
RIPA: radioimmunoprecipitation assay
RT: radiotherapy
SD: standard deviation
SDF: stromal cell-derived factor
SE: standard error
TAM: tumor-associated macrophage
TGF-β: transforming growth factor beta
TNF: tumor necrosis factor
TTP: time to progression

## References

[1] Polley MY, Lamborn KR, Chang SM, Butowski N, Clarke JL, Prados M (2010). Six-month progression-free survival as an alternative primary efficacy endpoint to overall survival in newly diagnosed glioblastoma patients receiving temozolomide. Neuro Oncol.

[2] Ringel F, Pape H, Sabel M, Krex D, Bock HC, Misch M, Weyerbrock A, Westermaier T, Senft C, Schucht P, Meyer B, Simon M, group SNs (2016). Clinical benefit from resection of recurrent glioblastomas: results of a multicenter study including 503 patients with recurrent glioblastomas undergoing surgical resection. Neuro Oncol.

[3] Seidlitz A, Siepmann T, Lock S, Juratli T, Baumann M, Krause M (2015). Impact of waiting time after surgery and overall time of postoperative radiochemotherapy on treatment outcome in glioblastoma multiforme. Radiat Oncol.

[4] Kesari S (2011). Understanding glioblastoma tumor biology: the potential to improve current diagnosis and treatments. Semin Oncol.

[5] Womeldorff M, Gillespie D, Jensen RL (2014). Hypoxia-inducible factor-1 and associated upstream and downstream proteins in the pathophysiology and management of glioblastoma. Neurosurg Focus.

[6] Lemée JM, Clavreul A, Menei P (2015). Intratumoral heterogeneity in glioblastoma: don’t forget the peritumoral brain zone. Neuro Oncol.

[7] Balasubramaniyan V, Vaillant B, Wang S, Gumin J, Butalid ME, Sai K, Mukheef F, Kim SH, Boddeke HW, Lang F, Aldape K, Sulman EP, Bhat KP (2015). Aberrant mesenchymal differentiation of glioma stem-like cells: implications for therapeutic targeting. Oncotarget.

[8] Zhou W, Bao S (2014). Reciprocal supportive interplay between glioblastoma and tumor-associated macrophages. Cancers (Basel).

[9] Zhou W, Ke SQ, Huang Z, Flavahan W, Fang X, Paul J, Wu L, Sloan AE, McLendon RE, Li X, Rich JN, Bao S (2015). Periostin secreted by glioblastoma stem cells recruits M2 tumour-associated macrophages and promotes malignant growth. Nat Cell Biol.

[10] Poli A, Wang J, Domingues O, Planaguma J, Yan T, Rygh CB, Skaftnesmo KO, Thorsen F, McCormack E, Hentges F, Pedersen PH, Zimmer J, Enger PO (2013). Targeting glioblastoma with NK cells and mAb against NG2/CSPG4 prolongs animal survival. Oncotarget.

[11] Cai J, Zhang W, Yang P, Wang Y, Li M, Zhang C, Wang Z, Hu H, Liu Y, Li Q, Wen J, Sun B, Wang X (2015). Identification of a 6-cytokine prognostic signature in patients with primary glioblastoma harboring M2 microglia/macrophage phenotype relevance. PLoS One.

[12] Barault L, Amatu A, Bleeker FE, Moutinho C, Falcomata C, Fiano V, Cassingena A, Siravegna G, Milione M, Cassoni P, De Braud F, Ruda R, Soffietti R (2015). Digital PCR quantification of MGMT methylation refines prediction of clinical benefit from alkylating agents in glioblastoma and metastatic colorectal cancer. Ann Oncol.

[13] Cabrini G, Fabbri E, Lo Nigro C, Dechecchi MC, Gambari R (2015). Regulation of expression of O6-methylguanine-DNA methyltransferase and the treatment of glioblastoma (Review). Int J Oncol.

[14] Champoux JJ (2001). DNA topoisomerases: structure, function, and mechanism. Annu Rev Biochem.

[15] Schoeffler AJ, Berger JM (2005). Recent advances in understanding structure-function relationships in the type II topoisomerase mechanism. Biochem Soc Trans.

[16] Raut LS (2015). Novel formulation of cytarabine and daunorubicin: A new hope in AML treatment. South Asian J Cancer.

[17] Teuffel O, Leibundgut K, Lehrnbecher T, Alonzo TA, Beyene J, Sung L (2013). Anthracyclines during induction therapy in acute myeloid leukaemia: a systematic review and meta-analysis. Br J Haematol.

[18] Mozdzanowska D, Wozniewski M (2015). Radiotherapy and anthracyclines - cardiovascular toxicity. Contemp Oncol (Pozn).

[19] Ezquer F, Gutierrez J, Ezquer M, Caglevic C, Salgado HC, Calligaris SD (2015). Mesenchymal stem cell therapy for doxorubicin cardiomyopathy: hopes and fears. Stem Cell Res Ther.

[20] Saleh EM (2015). Inhibition of topoisomerase IIalpha sensitizes FaDu cells to ionizing radiation by diminishing DNA repair. Tumour Biol.

[21] Kim JS, Kim SY, Lee M, Kim SH, Kim SM, Kim EJ (2015). Radioresistance in a human laryngeal squamous cell carcinoma cell line is associated with DNA methylation changes and topoisomerase II alpha. Cancer Biol Ther.

[22] Hayashi S, Hatashita M, Matsumoto H, Shioura H, Kitai R, Kano E (2006). Enhancement of radiosensitivity by topoisomerase II inhibitor, amrubicin and amrubicinol, in human lung adenocarcinoma A549 cells and kinetics of apoptosis and necrosis induction. Int J Mol Med.

[23] Chen Y, Lin TY, Chen JC, Yang HZ, Tseng SH (2006). GL331, a topoisomerase II inhibitor, induces radiosensitization of human glioma cells. Anticancer Res.

[24] Marin JJ, Briz O, Rodriguez-Macias G, Diez-Martin JL, Macias RI (2016). Role of drug transport and metabolism in the chemoresistance of acute myeloid leukemia. Blood Rev.

[25] Mechetner E, Kyshtoobayeva A, Zonis S, Kim H, Stroup R, Garcia R, Parker RJ, Fruehauf JP (1998). Levels of multidrug resistance (MDR1) P-glycoprotein expression by human breast cancer correlate with *in vitro* resistance to taxol and doxorubicin. Clin Cancer Res.

[26] Hawtin RE, Stockett DE, Byl JA, McDowell RS, Nguyen T, Arkin MR, Conroy A, Yang W, Osheroff N, Fox JA (2010a). Voreloxin is an anticancer quinolone derivative that intercalates DNA and poisons topoisomerase II. PLoS One.

[27] Hawtin RE, Stockett DE, Wong OK, Lundin C, Helleday T, Fox JA (2010). Homologous recombination repair is essential for repair of vosaroxin-induced DNA double-strand breaks. Oncotarget.

[28] Walsby EJ, Coles SJ, Knapper S, Burnett AK (2011). The topoisomerase II inhibitor voreloxin causes cell cycle arrest and apoptosis in myeloid leukemia cells and acts in synergy with cytarabine. Haematologica.

[29] Freeman C, Keane N, Swords R, Giles F (2013). Vosaroxin: a new valuable tool with the potential to replace anthracyclines in the treatment of AML?. Expert Opin Pharmacother.

[30] Hoch U, Lynch J, Sato Y, Kashimoto S, Kajikawa F, Furutani Y, Silverman JA (2009). Voreloxin, formerly SNS-595, has potent activity against a broad panel of cancer cell lines and *in vivo* tumor models. Cancer Chemother Pharmacol.

[31] Mjos KD, Cawthray JF, Jamieson G, Fox JA, Orvig C (2015). Iron(III)-binding of the anticancer agents doxorubicin and vosaroxin. Dalton Trans.

[32] Scatena CD, Kumer JL, Arbitrario JP, Howlett AR, Hawtin RE, Fox JA, Silverman JA (2010). Voreloxin, a first-in-class anticancer quinolone derivative, acts synergistically with cytarabine *in vitro* and induces bone marrow aplasia *in vivo*. Cancer Chemother Pharmacol.

[33] Advani RH, Hurwitz HI, Gordon MS, Ebbinghaus SW, Mendelson DS, Wakelee HA, Hoch U, Silverman JA, Havrilla NA, Berman CJ, Fox JA, Allen RS, Adelman DC (2010). Voreloxin, a first-in-class anticancer quinolone derivative, in relapsed/refractory solid tumors: a report on two dosing schedules. Clin Cancer Res.

[34] Krug LM, Crawford J, Ettinger DS, Shapiro GI, Spigel D, Reiman T, Temel JS, Michelson GC, Young DY, Hoch U, Adelman DC (2011). Phase II multicenter trial of voreloxin as second-line therapy in chemotherapy-sensitive or refractory small cell lung cancer. J Thorac Oncol.

[35] Lancet JE, Ravandi F, Ricklis RM, Cripe LD, Kantarjian HM, Giles FJ, List AF, Chen T, Allen RS, Fox JA, Michelson GC, Karp JE (2011). A phase Ib study of vosaroxin, an anticancer quinolone derivative, in patients with relapsed or refractory acute leukemia. Leukemia.

[36] Sasine JP, Schiller GJ (2015). Emerging strategies for high-risk and relapsed/refractory acute myeloid leukemia: novel agents and approaches currently in clinical trials. Blood Rev.

[37] Ravandi F, Ritchie EK, Sayar H, Lancet JE, Craig MD, Vey N, Strickland SA, Schiller GJ, Jabbour E, Erba HP, Pigneux A, Horst HA, Recher C (2015). Vosaroxin plus cytarabine versus placebo plus cytarabine in patients with first relapsed or refractory acute myeloid leukaemia (VALOR): a randomised, controlled, double-blind, multinational, phase 3 study. Lancet Oncol.

[38] Evanchik MJ, Allen D, Yoburn JC, Silverman JA, Hoch U (2009). Metabolism of (+)-1,4-dihydro-7-(trans-3-methoxy-4-methylamino-1-pyrrolidinyl)-4-oxo-1-(2-thiaz olyl)-1,8-naphthyridine-3-carboxylic acid (voreloxin; formerly SNS-595), a novel replication-dependent DNA-damaging agent. Drug Metab Dispos.

[39] Gordon IK, Graves C, Kil WJ, Kramp T, Tofilon P, Camphausen K (2012). Radiosensitization by the novel DNA intercalating agent vosaroxin. Radiat Oncol.

[40] Han MW, Lee JC, Choi JY, Kim GC, Chang HW, Nam HY, Kim SW, Kim SY (2014). Autophagy inhibition can overcome radioresistance in breast cancer cells through suppression of TAK1 activation. Anticancer Res.

[41] Sun Q, Liu T, Yuan Y, Guo Z, Xie G, Du S, Lin X, Xu Z, Liu M, Wang W, Yuan Q, Chen L (2015). MiR-200c inhibits autophagy and enhances radiosensitivity in breast cancer cells by targeting UBQLN1. Int J Cancer.

[42] Bienkowski M, Preusser M (2015). Prognostic role of tumour-infiltrating inflammatory cells in brain tumours: literature review. Curr Opin Neurol.

[43] Leroi N, Lallemand F, Coucke P, Noel A, Martinive P (2016). Impacts of ionizing radiation on the different compartments of the tumor microenvironment. Front Pharmacol.

[44] Stavrovskaya AA, Shushanov SS, Rybalkina EY (2016). Problems of glioblastoma multiforme drug resistance. Biochemistry (Mosc).

[45] Stapleton S, Jaffray D, Milosevic M (2017). Radiation effects on the tumor microenvironment: Implications for nanomedicine delivery. Adv Drug Deliv Rev.

[46] Tang B, Wu W, Wei X, Li Y, Ren G, Fan W (2014). Activation of glioma cells generates immune tolerant NKT cells. J Biol Chem.

[47] Dello Russo C, Lisi L, Tentori L, Navarra P, Graziani G, Combs CK (2016). Exploiting microglial functions for the treatment of glioblastoma. Curr Cancer Drug Targets.

[48] Jablonski KA, Amici SA, Webb LM, de Ruiz-Rosado J D, Popovich PG, Partida-Sanchez S, Guerau-de-Arellano M (2015). Novel markers to delineate murine M1 and M2 macrophages. PLoS One.

[49] Lancet JE, Roboz GJ, Cripe LD, Michelson GC, Fox JA, Leavitt RD, Chen T, Hawtin R, Craig AR, Ravandi F, Maris MB, Stuart RK, Karp JE (2015). A phase 1b/2 study of vosaroxin in combination with cytarabine in patients with relapsed or refractory acute myeloid leukemia. Haematologica.

[50] Stuart RK, Cripe LD, Maris MB, Cooper MA, Stone RM, Dakhil SR, Turturro F, Stock W, Mason J, Shami PJ, Strickland SA, Costa LJ, Borthakur G (2015). REVEAL-1, a phase 2 dose regimen optimization study of vosaroxin in older poor-risk patients with previously untreated acute myeloid leukaemia. Br J Haematol.

[51] Thacker J, Ganesh AN (1990). DNA-break repair, radioresistance of DNA synthesis, and camptothecin sensitivity in the radiation-sensitive irs mutants: comparisons to ataxia-telangiectasia cells. Mutat Res.

[52] Nitiss JL (2009). DNA topoisomerase II and its growing repertoire of biological functions. Nat Rev Cancer.

[53] Hong Y, Sang M, Shang C, Xue YX, Liu YH (2012). Quantitative analysis of topoisomerase II alpha and evaluation of its effects on cell proliferation and apoptosis in glioblastoma cancer stem cells. Neurosci Lett.

[54] Desmarais G, Charest G, Fortin D, Bujold R, Mathieu D, Paquette B (2015). Cyclooxygenase-2 inhibitor prevents radiation-enhanced infiltration of F98 glioma cells in brain of Fischer rat. Int J Radiat Biol.

[55] Han S, Liu Y, Li Q, Li Z, Hou H, Wu A (2015). Pre-treatment neutrophil-to-lymphocyte ratio is associated with neutrophil and T-cell infiltration and predicts clinical outcome in patients with glioblastoma. BMC Cancer.

[56] Cusulin C, Chesnelong C, Bose P, Bilenky M, Kopciuk K, Chan JA, Cairncross JG, Jones SJ, Marra MA, Luchman HA, Weiss S (2015). Precursor states of brain tumor initiating cell lines are predictive of survival in xenografts and associated with glioblastoma subtypes. Stem Cell Reports.

[57] Gil-Ranedo J, Mendiburu-Elicabe M, Garcia-Villanueva M, Medina D, del Alamo M, Izquierdo M (2011). An off-target nucleostemin RNAi inhibits growth in human glioblastoma-derived cancer stem cells. PLoS One.

[58] Gravina GL, Mancini A, Scarsella L, Colapietro A, Jitariuc A, Vitale F, Marampon F, Ricevuto E, Festuccia C (2016). Dual PI3K/mTOR inhibitor, XL765 (SAR245409), shows superior effects to sole PI3K [XL147 (SAR245408)] or mTOR [rapamycin] inhibition in prostate cancer cell models. Tumour Biol.

[59] Gravina GL, Marampon F, Sherris D, Vittorini F, Di Cesare E, Tombolini V, Lenzi A, Jannini EA, Festuccia C (2014). Torc1/Torc2 inhibitor, Palomid 529, enhances radiation response modulating CRM1-mediated survivin function and delaying DNA repair in prostate cancer models. Prostate.

[60] Solberg TD, Iwamoto KS, Norman A (1992). Calculation of radiation dose enhancement factors for dose enhancement therapy of brain tumours. Phys Med Biol.

[61] Festuccia C, Gravina GL, D’Alessandro AM, Muzi P, Millimaggi D, Dolo V, Ricevuto E, Vicentini C, Bologna M (2009). Azacitidine improves antitumor effects of docetaxel and cisplatin in aggressive prostate cancer models. Endocr Relat Cancer.

[62] Gravina GL, Mancini A, Marampon F, Colapietro A, Delle Monache S, Sferra R, Vitale F, Richardson PJ, Patient L, Burbidge S, Festuccia C (2017). The brain-penetrating CXCR4 antagonist, PRX177561, increases the antitumor effects of bevacizumab and sunitinib in preclinical models of human glioblastoma. J Hematol Oncol.

[63] Bruzzese F, Di Gennaro E, Avallone A, Pepe S, Arra C, Caraglia M, Tagliaferri P, Budillon A (2006). Synergistic antitumor activity of epidermal growth factor receptor tyrosine kinase inhibitor gefitinib and IFN-alpha in head and neck cancer cells *in vitro* and *in vivo*. Clin Cancer Res.

[64] Reynolds CP, Sun BC, DeClerck YA, Moats RA, Blumenthal RD (2004). Assessing Growth and Response to Therapy in Murine Tumor Models. Chemosenstitivity: Volume II: *In vivo* Models, Imaging, and Molecular Regulators.

[65] Kemper EM, Leenders W, Kusters B, Lyons S, Buckle T, Heerschap A, Boogerd W, Beijnen JH, van Tellingen O (2006). Development of luciferase tagged brain tumour models in mice for chemotherapy intervention studies. Eur J Cancer.

[66] Breton E, Goetz C, Kintz J, Accart N, Aubertin G, Grellier B, Erbs P, Rooke R, Constantinesco A, Choquet P (2010). *In vivo* preclinical low-field MRI monitoring of tumor growth following a suicide-gene therapy in an orthotopic mice model of human glioblastoma. C R Biol.

