# Supplementary material for: Enhancement of radiosensitivity by the novel anticancer quinolone derivative vosaroxin in preclinical glioblastoma models
Source: Oncotarget. 2017 Mar 13;8(18):29865–86. doi: 10.18632/oncotarget.16168 (PMC5444710; doi:10.18632/oncotarget.16168)
Supplement: Supplementary file 1 [file oncotarget-08-29865-s001.pdf]

Enhancement of radiosensitivity by the novel anticancer quinolone derivative vosaroxin in preclinical glioblastoma models

Supplementary Materials

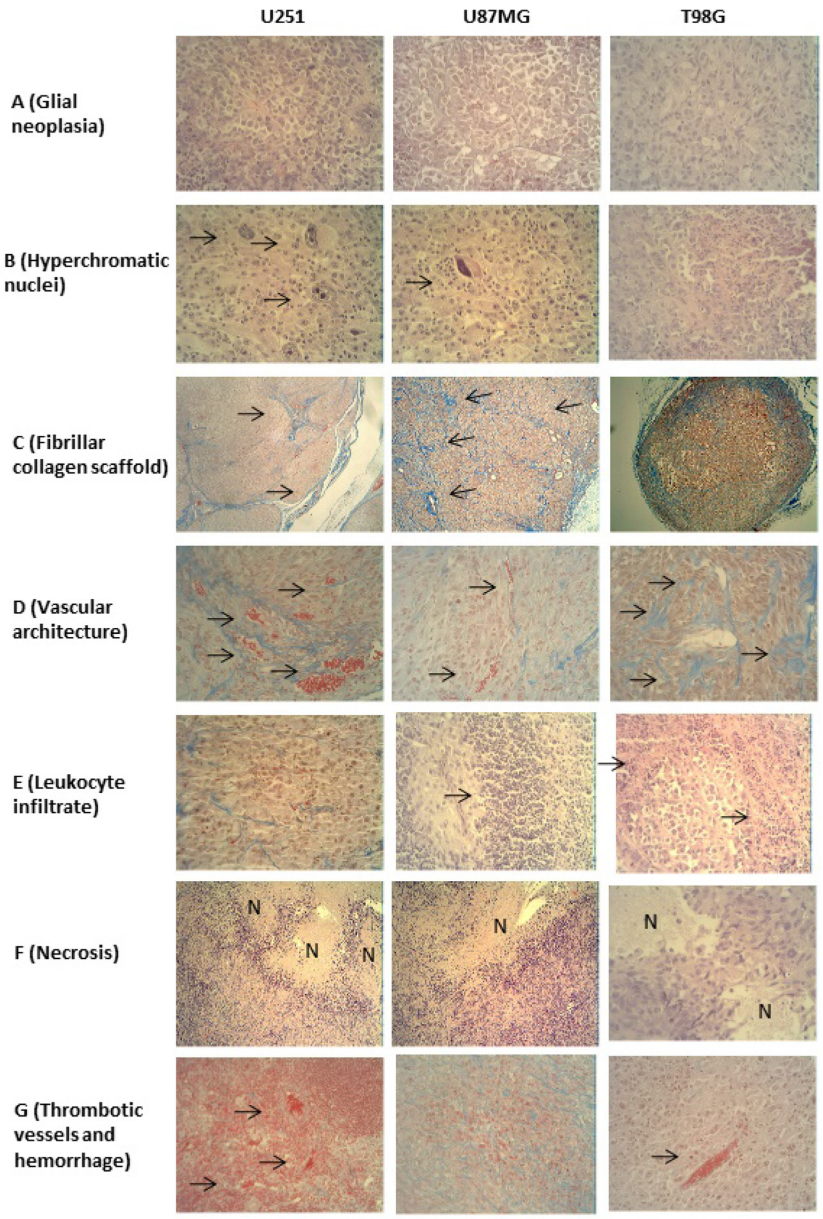

Supplementary Figure 1: Histopathologic appearance of experimental gliomas. Histopathologic analysis of xenograft tumors (derived from U251, U87MG, and T98G cells) showed the following morphologic characteristics, as indicated by arrows: (**A**) tightly packed sheets of heterogeneous tumor cell population, which resulted in round to polygonal cell morphology, or (**B**) spindle-shaped morphology with abundant intensely eosinophilic cytoplasm and hyperchromatic nuclei and nucleoli. Tumor cells were dispersed on a fibrillar collagen background (**C**), which enveloped abundant vasculature (**D**). Leukocyte infiltrate surrounding the tumor (**E**). Tissues with pseudopalisading necrosis ("N") (**F**), and thrombotic vessels and hemorrhage (**G**).

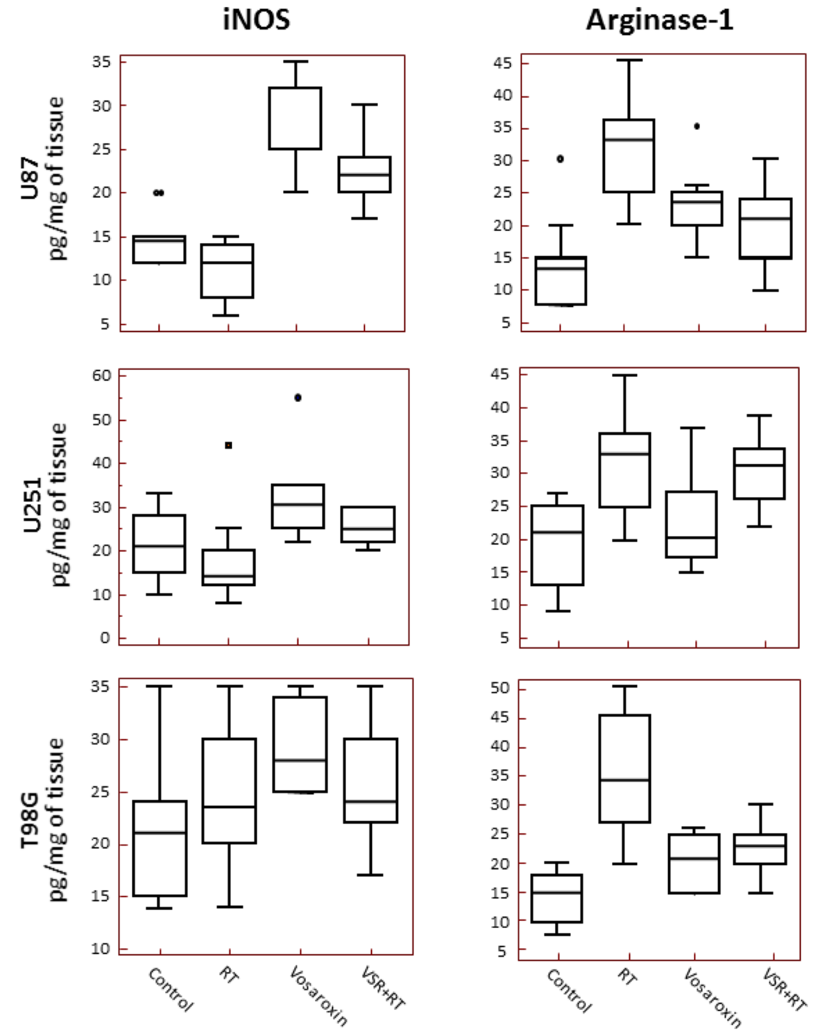

Supplementary Figure 2: ELISA assays of iNOS and arginase-1 from tissue extracts. Expression levels of iNOS and arginase-1 as markers for M1 and M2 macrophages as determined in tissue extracts from U87, U251, and T98G xenografts after RT, vosaroxin, or vosaroxin + RT treatments.

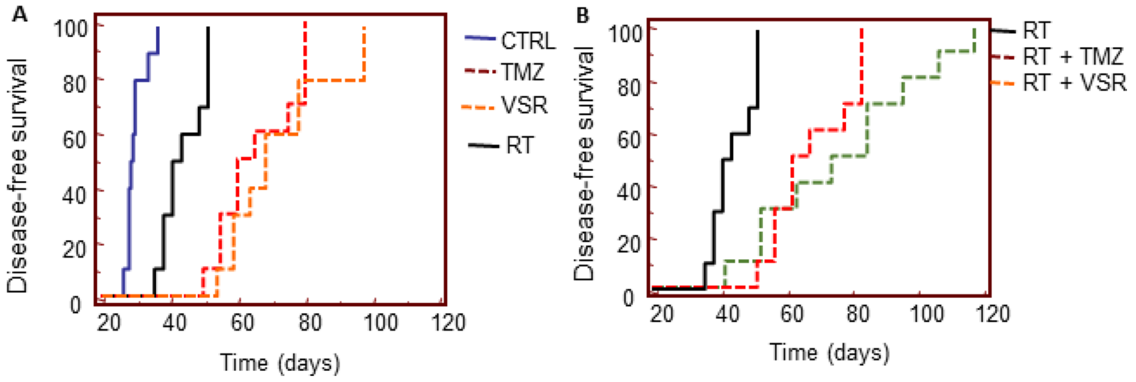

Supplementary Figure 3: *In vivo* experiments: orthotopic intrabrain model. (**A**) Comparison of the effects of single treatments versus control (CTRL) on disease-free survival. (**B**) Comparison of the radiosensitizing effects of temozolomide (TMZ) and vosaroxin (VSR) on disease-free survival.

MATERIALS AND METHODS

Gravina et al. Enhancement of radiosensitivity by the novel anticancer quinolone derivative vosaroxin in preclinical glioblastoma models

Supplementary Table 1: Hazard ratios for probability of tumor progression in xenograft models (additional comparisons)

Treatments Compared	U87MG		U251		T98G	
	HR (95% CI)	*P* Value	HR (95% CI)	*P* Value	HR (95% CI)	*P* Value
CTRL vs RT	4.2 (1.4–12.7)	< 0.0001	3.1 (1.1–8.7)	0.0012	2.7 (1.0–7.4)	0.0062
CTRL vs VSR	4.6 (1.5–14.5)	< 0.0001	4.2 (1.4–12.7)	< 0.0001	4.1 (1.4–12.6)	< 0.0001
CTRL vs TMZ	4.6 (1.2–11.5)	< 0.0001	3.9 (1.3–11.5)	0.0001	1.3 (0.6–3.2)	0.41 (NS)
RT vs VSR	3.5 (1.4–11.2)	< 0.0001	3.2 (1.1–9.0)	0.0009	4.6 (1.5–14.3)	< 0.0001
RT vs TMZ	3.3 (1.5–13.0)	< 0.0001	2.3 (1.1–6.1)	0.0244	1.9 (0.8–4.8)	0.06 (NS)
CTRL vs VSR +RT	7.8 (2.8–23.6)	< 0.0001	4.7 (1.5–14.9)	< 0.0001	5.1 (1.6–16.2)	< 0.0001
CTRL vs TMZ + RT	6.0 (2.1–21.0)	< 0.001	5.5 (1.7–17.8)	< 0.0001	3.7 (1.3–8.0)	< 0.0001
RT vs TMZ + RT	3.7 (1.3–10.9)	0.0030	5.0 (1.6–15.8)	< 0.0001	3.3 (1.2–9.4)	0.0005
TMZ vs TMZ + RT	1.7 (1.2–3.3)	0.09 (NS)	4.6 (1.5–14.2)	< 0.0001	2.5 (1.0–6.7)	0.0097

CI: confidence interval; CTRL: vehicle control; HR: hazard ratio; RT: radiotherapy; TMZ: temozolomide; VSR: vosaroxin.

Supplementary Table 2: Hazard ratios for disease-free survival and overall survival in orthotopic U251 models (additional comparisons)

Treatments Compared	Disease–Free Survival		Overall Survival	
	HR (95% CI)	*P* Value	HR (95% CI)	*P* Value
CTRL vs RT	4.0 (1.3–15.0)	< 0.0001	4.3 (1.4–12.7)	0.0091
CTRL vs VSR	5.7 (2.5–16.2)	< 0.0001	10.2 (3.0–35.1)	0.0002
CTRL vs TMZ	5.0 (1.9–15.2)	< 0.0001	8.1 (2.5–25.7)	0.0006
RT vs VSR	6.8 (2.0–23.0	< 0.0001	5.5 (1.2–12.5)	0.0015
RT vs TMZ	2.5 (1.0–6.7)	0.0123	5.9 (1.6–21.4)	0.0019
CTRL vs VSR + RT	7.5 (3.0–16.7)	< 0.0001	14.5 (3.6–38.4)	< 0.0001
CTRL vs TMZ + RT	6.5 (1.7–14.8)	< 0.0001	8.4 (2.5–27.9)	0.0004
RT vs TMZ + RT	3.6 (1.2–12.5)	0.0023	3.2 (1.1–9.0)	0.0329
TMZ vs TMZ + RT	2.6 (1.2–8.2)	< 0.0001	3.9 (1.2–12.1)	0.0179

CI: confidence interval; CTRL: vehicle control; HR: hazard ratio; RT: radiotherapy; TMZ: temozolomide; VSR: vosaroxin.
